# Competitive Charge Separation Pathways in a Flexible Molecular Folda-Dimer

**DOI:** 10.1021/acs.jpcb.3c07134

**Published:** 2024-02-10

**Authors:** Kalyani Thakur, Saptarshi Datta, Paul W. M. Blom, Debangshu Chaudhuri, Charusheela Ramanan

**Affiliations:** † Max Planck Institute for Polymer Research, Ackermannweg 10, Mainz 55128, Germany; ‡ Department of Chemical Sciences, Indian Institute of Science Education and Research (IISER) Kolkata, Mohanpur 741246, India; § Department of Physics and Astronomy, Vrije Universiteit Amsterdam, De Boelelaan 1081, 1081HV Amsterdam, Netherlands

## Abstract

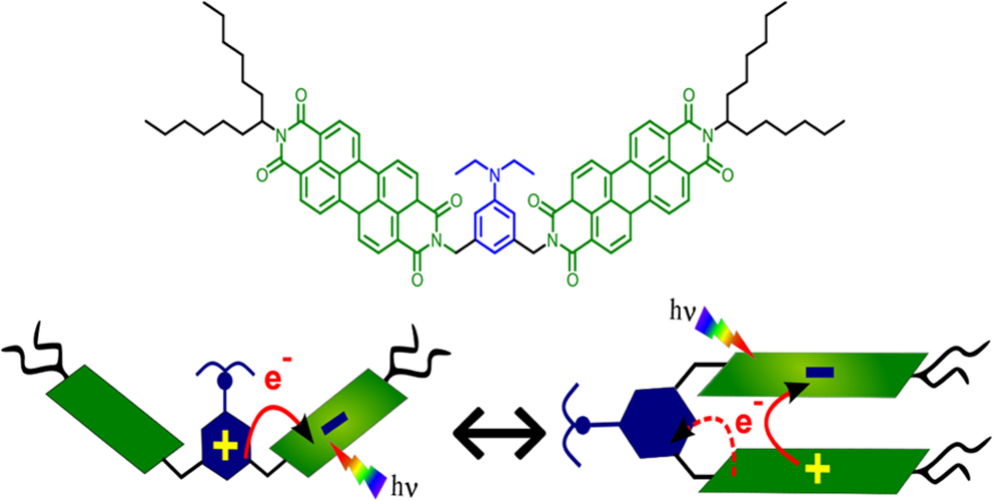

We report the photophysical properties of a molecular folda-dimer system **PDI-AnEt**_**2**_**-PDI**, where the electron-donating *N*,*N*-diethylaniline (AnEt_2_) moiety bridges two electron-accepting perylene diimide (PDI) chromophores. The conformationally flexible **PDI-AnEt**_**2**_**-PDI** adopts either an open (two PDIs far apart) or folded (two PDIs within π-stacking distance) conformation, depending on the solvent environment. We characterized the photoinduced charge separation dynamics of both open and folded forms in solvents of varying polarity. The open form undergoes charge separation to give **PDI^•–^-AnEt_2_^•+^-PDI** (Bridge electron transfer) independent of solvent polarity. The folded form exhibits two charge separation photoproducts, yielding both **PDI^•–^-AnEt_2_^•+^-PDI** and **PDI^•–^-AnEt_2_-PDI^•+^**, the latter of which is formed via symmetry-breaking charge separation (SBCS) between the two π-stacked PDI chromophores. Our results further indicate that the conformational flexibility of the folda-dimer leads to unexpected excimer formation in some open form conditions. In contrast, no excimer formation is observed in the folded form, indicating that this geometry preferentially yields the SBCS instead. Our results provide insight into how conformationally flexible folda-dimer systems can be designed and built to tune competitive photophysical pathways.

## Introduction

Molecular chromophore systems are promising candidates for light-activated applications, such as optoelectronics, photocatalysis, sensing, and imaging.^1−5^ These materials exhibit excellent light-harvesting characteristics and synthetic tunability, but they also demonstrate very complicated potential energy surfaces with multiple competitive photoactive pathways. Electronic and vibrational coupling between adjacent chromophores mediates light-driven processes, such as photoinduced charge separation, light-harvesting, excitation energy transfer, and singlet fission. There is a significant need and interest to understand the interplay between molecular conformation and photophysics within such complex excited state energy surfaces.

Electronic and vibrational coupling between molecular chromophores occurs through covalent and noncovalent interactions. In particular, π-stacking between adjacent molecules can alter the potential energy surface relative to the monomeric condition.^6−8^ This is critical when considering solid-state applications, such as organic photovoltaics and light-emitting diodes, where π-stacking interactions between a large number of chromophores can result in the formation of dark trap states that hinder device performance. This behavior is even observed in smaller-scale dimer systems, which exhibit π-stacking chromophore association. While photoexcited dynamics in such materials are often described as a sequence of well-defined (diabatic) electronic states, these states are often coherently mixed with varying electronic and spin character.^9−11^ Such coherent mixing indicates that the involved states are similar in both energy and geometry. This also means that the system is rarely in a “pure” Frenkel exciton state, but more likely in a mixture, for example, between a Frenkel and charge transfer state.^12−14^ The ultimate photoexcited dynamics of a given system will depend therefore on how the molecular conformation and solvent environment tunes the potential energy surface and electronic state mixing and directs the evolution of the photoexcited population.

**Figure 1:**
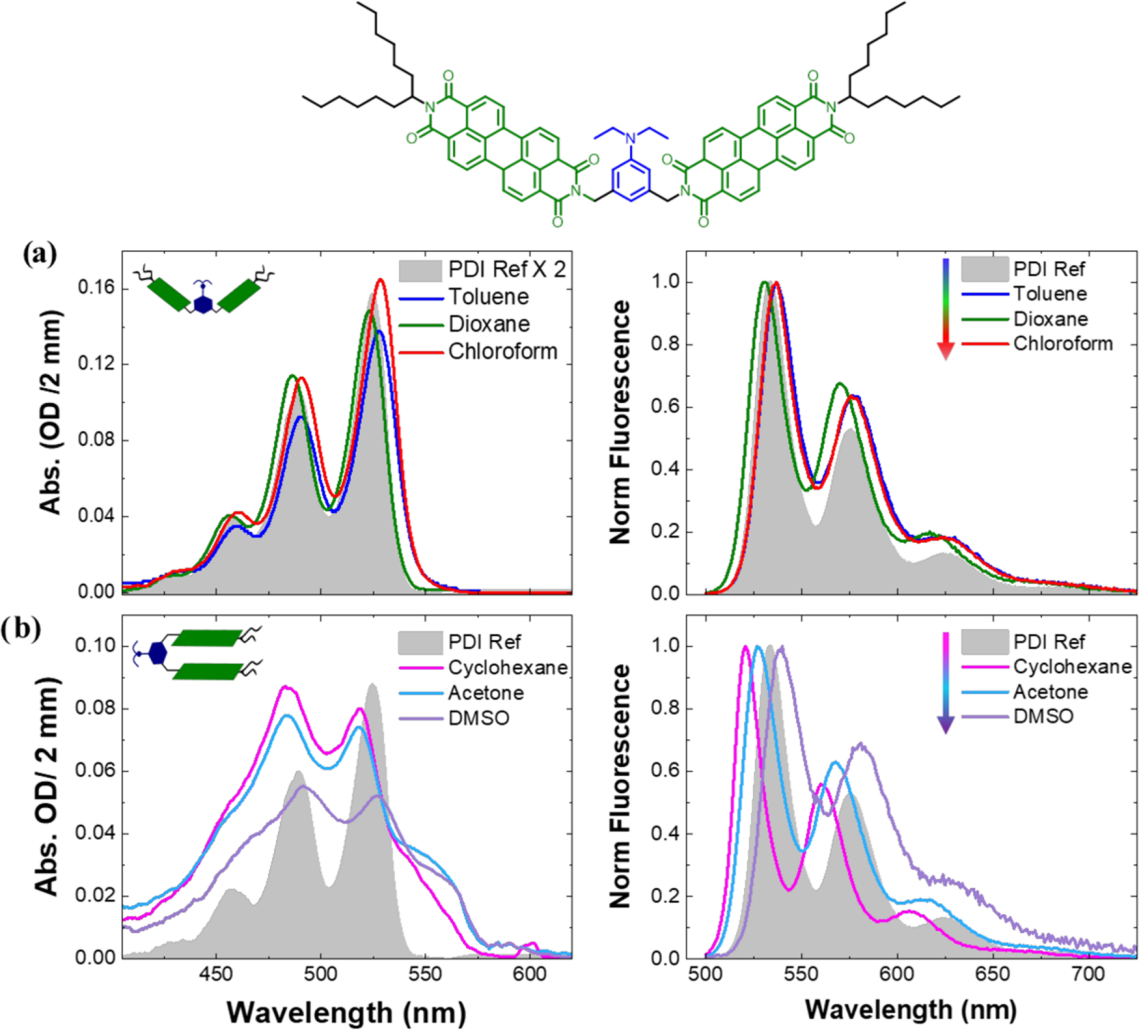
Molecular structure of acceptor–donor–acceptor **PDI-AnEt_2_-PDI** along with steady-state absorption (left) and fluorescence (right) in the (a) open form and (b) folded form, each in three solvents of varying polarity. Absorbance in OD is measured at 6 uM. The arrow indicates increasing solvent polarity, starting from blue (pink) as the least polar solvent and red (purple) as the most polar solvent. The molecular structure of PDI-ref is shown in Figure S14.

Structurally well-defined bi- and trichromophoric systems have often been used as model systems to illustrate the role of structure on the exciton dynamics in molecular assemblies.^15−20^ Many such model systems studied in the past had a rigid molecular framework. The covalent bridge between the chromophoric units in those systems did not offer any structural flexibility, thus fixing the relative orientations and electronic coupling between the chromophores. Conformationally dynamic molecular systems, on the other hand, offer versatile frameworks to develop responsive materials with self-assembling characteristics. A flexible design allows insight into the interplay between the structural dynamics of a molecular assembly and exciton photophysics. We designed a folda-dimeric system **PDI-AnEt**_**2**_**-PDI** (see Figure 1 for structure) wherein two electron-accepting perylene diimide (PDI) chromophores are covalently bound to an electron-donating *N*,*N*-diethylaniline (AnEt_2_). The AnEt_2_ also serves as a bridging moiety, imparting conformational flexibility to the system, thus giving the opportunity to investigate donor–acceptor interaction in varying molecular orientations. PDI chromophores offer an attractive model system to study the correlation between intermolecular coupling, conformation, and photophysics. They are chemically robust, exhibit high extinction coefficients in the visible region, and are also good electron acceptors and transporters.^1,21^ The last of these is facilitated by the tendency of PDI chromophores to form π-stacked architectures, for example, as H-type or J-type aggregates.^22−25^

Depending on the solvent environment, **PDI-AnEt**_**2**_**-PDI** adopts either an open conformation, with the two PDI chromophores far apart from each other, or a folded configuration, with the two PDI chromophores within the π-stacking distance. This allows us to study the dependence of photophysical dynamics on the folda-dimer conformation. We find that the folda-dimer exhibits multiple simultaneous photophysical pathways. In the open form, charge separation with the bridging AnEt_2_ is observed, which we term Bridge electron transfer (Bridge ET). In the folded form, Bridge ET competes with a second charge separation pathway of symmetry-breaking charge separation (SBCS) between the two π-stacked PDI chromophores. Furthermore, our findings indicate that SBCS outcompetes any excimer formation in the folded form. The conformational flexibility of **PDI-AnEt**_**2**_**-PDI** further controls the photophysics by allowing for dynamic structural changes. Our results yield insight into how to mediate competitive photophysical pathways and mixed-electronic states in conformationally dynamic molecular chromophore systems.

## Experimental Details

### Materials

The PDI monomer for control experiments (*N*,*N*-dipentyl-3,4,9,10-perylenedicarboximide, referred to as **PDI-ref**) and all of the solvents used in this study were purchased from Sigma-Aldrich and used without further purification. Details of synthetic procedures and characterization (NMR, CV) of **PDI-AnEt**_**2**_**-PDI** are given in the Supporting Information.

### Steady-State Absorption and Fluorescence

Steady-state absorption spectra were collected by using a halogen-deuterium lamp (DH2000-DUV, OceanOptics) connected to a USB spectrometer (34000-UV–vis-EIS, OceanOptics). Steady-state fluorescence was measured in a Horiba/Jobin Yvon Fluorolog-3 spectrofluorometer using an excitation wavelength of 440 nm. Quantum yield is calculated relative to a reference PDI chromophore from the literature.^26^

### Transient Absorption Spectroscopy

TA spectroscopy was measured using a Helios-Fire pump–probe setup (Ultrafast Systems). This is paired with a regeneratively amplified 1030 nm laser (Light Conversion, Pharos, 200 fs, 200 uJ), set at an effective repetition rate of 1 kHz via an internal pulse picker. A small portion (20%) of the 1030 nm fundamental is directed toward an optical delay line and, subsequently, focused onto a sapphire crystal to generate the broadband probe light (480–900 nm). The remaining 80% of the 1030 nm fundamental is fed to an optical parametric amplifier (Light Conversion, Orpheus-F) to generate the pump light. Samples were photoexcited (pumped) at λ_ex_ = 490 and 530 nm for various experiments, as detailed further in the results. Samples were measured at 6 μM concentration in 2 mm path length cuvettes with continuous stirring during the experiment. The excitation energy was adjusted to 100 nJ/pulse for all experiments. The relative polarization between the pump and probe beams was set at 54.7° to avoid anisotropic effects.

Global analysis of the TA data was done using the R-package TIMP software^27^ with the graphical interface Glotaran (v.1.5.1).^28^ Global analysis is a method where all of the wavelengths are fit simultaneously to a set of common time constants representing a sum of exponential decay.^29^ This fitting scheme allows us to analyze our data using both sequentially interconverting and parallel decaying sum-of-exponential models. The sequential model results in a number of evolution-associated difference spectra (EADS) converting into successive EADS with associated monoexponential rates. This method quantitatively describes the system’s evolution of excited and intermediate states. The parallel model produces decay-associated difference spectra (DADS). The DADS represent spectral changes related to the specific time constant and are equivalent to the spectral change occurring during the evolution of one EADS to the next. The global analysis scheme evaluates EADS and DADS simultaneously. Since both methods are mathematically equal, the same time constant applies to both EADS and DADS. While sequential EADS are most commonly seen to interpret TA data, it is important to note that the spectral line shape of EADS does not always reflect the pure electronic states and may be difficult to interpret when kinetically competitive processes are occurring. The DADS can offer more insight in the case where multiple processes occur on similar time scales. In this work, the results are interpreted using DADS due to possible branching and kinetic competition between photophysical pathways.

### Time-Resolved Photoluminescence

TRPL was measured using a streak camera system (C5680, Hamamatsu). The samples were photoexcited at λ_ex_ = 440 nm, using the output from a regeneratively amplified Ti/sapphire laser (Coherent Astrella, 5 mJ, 35 fs, 800 nm, 1 kHz) paired with an optical parametric amplifier (Coherent Opera Solo). The samples were measured in 2 mm path length cuvettes with front-face illumination. The temporal range of the measurement was adjusted based on solvent: DMSO (10 ns time window, 0.12 ns instrument response) and other solvents (20 ns time window, 0.24 ns instrument response), with an additional measurement for chloroform as noted in the text (50 ns time window, 0.5 ns instrument response).

## Results and Discussion

### Solvent-Dependent Molecular Conformation

Figure 1 shows the steady-state absorption spectra of **PDI-AnEt**_**2**_**-PDI** in six different solvents with varying solvent polarity and dielectric constant (Table S4), along with a comparison to **PDI-ref**, a PDI monomer used as a control, measured in toluene (see Figure S14 for the molecular structure). The absorption spectrum of **PDI-ref** in gray consists of three vibronic peaks associated with the 0–0, 0–1, and 0–2 transitions at 530, 490, and 460 nm, respectively.^1,26^ The absorption spectra for **PDI-AnEt**_**2**_**-PDI** in chloroform, dioxane, and toluene (Figure 1a, left) match closely those of **PDI-ref**. In contrast, in the absorption spectra of **PDI-AnEt**_**2**_**-PDI** in DMSO, acetone, and cyclohexane (Figure 1b, left), the aforementioned three characteristic peaks are blue-shifted, and the relative intensities of the 0–0 and 0–1 vibronic bands are inverted. These changes are consistent with H-type coupling between PDI chromophores.^13,22,30^ The similarity to **PDI-ref** in chloroform, dioxane, and toluene indicates that, in these solvents, **PDI-AnEt**_**2**_**-PDI** is in the open form, with the two PDI chromophores sufficiently far away to avoid any through-space electronic coupling with each other. The characteristic H-type spectra in DMSO, acetone, and cyclohexane indicate the presence of PDI π-stacking. As all samples are measured at the same concentration (6 μM), it is unlikely that the H-type spectra in these solvents are due to intermolecular aggregation.^31^ Therefore, in these solvents, the two PDI chromophores fold toward each other around the anchor point of the bridging AnEt_2_ and equilibrate within the π-stacking distance, yielding the folded form. The trends observed are consistent with the previously reported binding energies for PDI π-stacking (Table S1). These literature studies demonstrate that PDI π-stacking depends not only on solvent polarity, but also electrostatic interactions between chromophores,^32,33^ Chloroform, dioxane, and toluene all exhibit similar stacking association constants (ca. −15 kJ/mol), while the stacking energies in DMSO, acetone, and cyclohexane are all more negative (between −17 and −30 kJ/mol). Previous work has reported preferential folding in DMSO for a PDI folda-dimer,^34^ and the stronger binding energies in acetone and cyclohexane further support that the H-type spectra are due to folding, and not intermolecular aggregation. Concentration-dependent steady-state measurements (Figure S10) also show minimal changes to the optical characteristics upon dilution, supporting the conclusion that the chromophoric coupling is intramolecular in nature. The H-type spectra in Figure 1b also demonstrate a red-shifted shoulder at ∼560 nm in addition to the expected blue shift. Such a feature is attributed to rotationally displaced/twisted stacking between adjacent chromophores,^31,35,36^ leading to an increased contribution from the low Davydov component to the absorption spectrum. The appearance of this feature in the folded PDI-AnEt_2_-PDI suggests that the chromophores adopt such a twist to accommodate the alkyl chain substituents.

The steady-state fluorescence (Figure 1, right) offers further insight into the molecular conformation of **PDI-AnEt**_**2**_**-PDI** and its effect on the optical properties. In the open form, **PDI-AnEt**_**2**_**-PDI** exhibits similar PL spectra as **PDI-ref**, with three characteristic peaks between 540–620 nm and mirror image symmetry to the absorption spectrum.^26^ The fluorescence spectrum of **PDI-AnEt**_**2**_**-PDI** in dioxane is slightly blue-shifted relative to that in chloroform or toluene. The fluorescence spectra of folded **PDI-AnEt**_**2**_**-PDI** (DMSO, acetone, cyclohexane) also exhibit similar lineshapes to that of **PDI-ref**, despite the evidence of H-stacking in the absorption spectra. The emission of H-stacked PDI is expected to be red-shifted relative to the monomer, with a broad Gaussian-type line shape, due to the formation of an excimer-like state upon photoexcitation.^23,37^ The fact that the fluorescence spectra observed in the folded form follow a line shape similar to **PDI-ref** is presumably from a small fraction of unfolded dimer. Furthermore, we see a relative increase in the intensity of the lower-energy peaks, most strikingly for DMSO. This is consistent with the presence of an underlying additional peak contributing to the fluorescence line shape. This could be from an excimeric and/or charge transfer (CT) state contribution. The enhanced Stokes shift in the folded form fluorescence spectra with increasing solvent polarity supports a CT contribution to the emission.^38,39^ Previous work has shown that in other cofacial PDI stacks, excited states are not purely Frenkel or CT, but a mixture of (quasi)adiabatic states, and we propose a similar behavior for the folded form of **PDI-AnEt**_**2**_**-PDI**.^40^

### Fluorescence Quantum Yield Increases Upon Protonation

**Figure 2:**
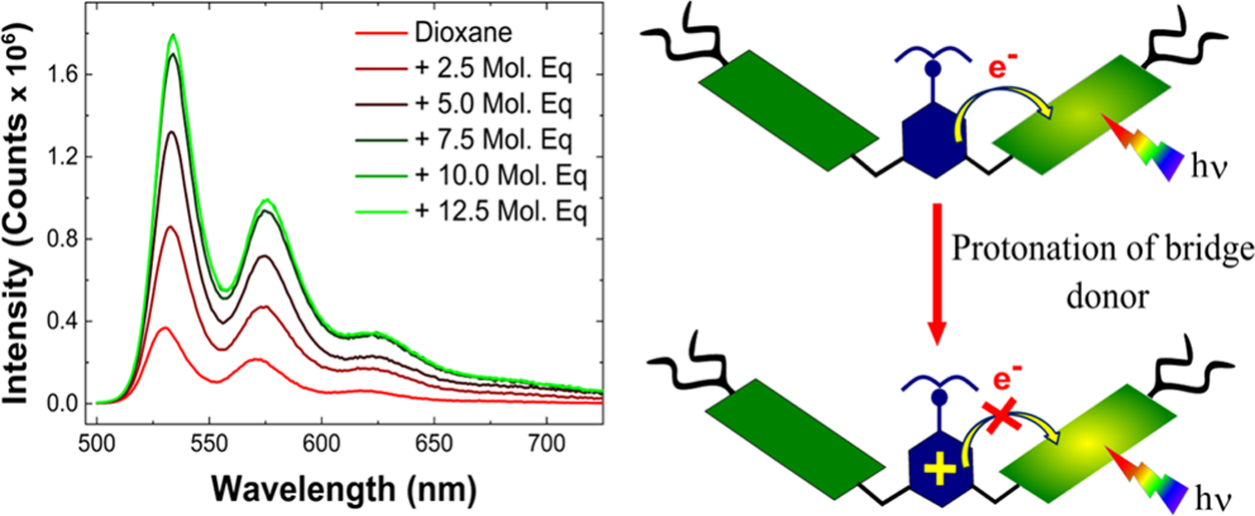
PDI-AnEt_2_-PDI in dioxane shows an increase in PDI fluorescence intensity with the addition of HCl, saturating at 10 mol equiv. This is in agreement with the proposed photoinduced Bridge ET: the acid protonates the bridging AnEt_2_ donor, blocking the charge separation pathway, and thus increasing the observed PDI fluorescence.

The fluorescence quantum yield of **PDI-AnEt**_**2**_**-PDI** is quenched relative to that of **PDI-ref** (Table 1). This is consistent with a new channel for photoexcited population decay in **PDI-AnEt**_**2**_**-PDI**. The folda-dimer is designed to undergo photoinduced charge separation between the electron-donating AnEt_2_ bridge and one of the electron-accepting PDI chromophores (Bridge ET). We carried out a protonation experiment to further confirm this hypothesis. Figure 2 shows the fluorescence spectrum of **PDI-AnEt**_**2**_**-PDI** in dioxane with increasing molar equivalents of HCl. Dioxane is chosen for this experiment, as it has good miscibility with the aqueous HCl solution. The absolute fluorescence intensity of **PDI-AnEt**_**2**_**-PDI** increases with each acid addition step and saturates at four times the original intensity upon the addition of 10 mol equivalent of acid. This finding further supports a photoexcited interaction between bridging AnEt_2_ and PDI ligands. Upon photoexcitation of **PDI-AnEt**_**2**_**-PDI**, electron transfer would quench the PDI fluorescence. The acid protonates the AnEt_2_ moiety, blocking the electron transfer pathway between the PDI acceptor and the bridge donor. However, other relaxation processes could also lead to fluorescence quenching such as the aforementioned possible excimer formation in the folded configuration. Furthermore, as seen in Table 1, the QY changes depending on the solvent environment. In chloroform, the QY is 49% relative to that of **PDI-ref**, but the value drops to much lower values of 6–7% in dioxane and toluene. In protonated dioxane, the QY is recovered to near that of chloroform (46%). In folded **PDI-AnEt**_**2**_**-PDI**, the values are consistently low (3–8%) and drop to the lowest in DMSO. The drop in QY relative to **PDI-ref** is consistent with the formation of a photoproduct, such as charge separation or excimer formation, competing with the fluorescence decay pathway from the singlet excited state. We further characterized the fluorescence decay dynamics of **PDI-AnEt**_**2**_**-PDI** in each of the solvents using time-resolved photoluminescence (TRPL). Selected PL spectra and decay kinetics, along with fits, are shown in Figures S12 and S13. The PL decay kinetics for all solvents are fit to a decay lifetime of approximately 4 ns with the exception of DMSO, which exhibits a much faster decay of 1.2 ns. The PL spectral lineshapes, as in the steady-state PL, match that of the PDI monomer in all solvents, with no significant excimer contribution noted. An H-stacked excimer would yield an emissive profile red-shifted relative to the monomer,^24^ and this characteristic can also be examined by comparing the PL decay kinetics at the 0–0 and 0–1 emission bands. An excimeric contribution would yield a much longer lifetime at the 0–0 vs the 0–1 band. The comparison of decay kinetics in all solvent conditions indicates a slight increase in PL decay at the 0–1 band, suggesting some possible small excimer contribution. However, this could not be temporally distinguished in the fitting. These solvent-dependent differences in PL QY and decay dynamics along with the known change of molecular configuration between open and folded form with solvent suggest that additional processes besides the Bridge ET may also be playing a role in the photoexcited dynamics of **PDI-AnEt**_**2**_**-PDI**.

**Table 1:** Fluorescence Quantum Yield of **PDI-AnEt**_**2**_**-PDI** in the Various Solvents, Determined in Reference to **PDI-Ref**^a^

configuration	solvent	lifetime (TRPL) (ns)	QY (%)
open	chloroform	3.9	49
	dioxane/dioxane(+)^a^	4.1	7.5/(46)
	toluene	4.4	6.7
folded	DMSO	1.2	3.3
	acetone	4.5	8.2
	cyclohexane	4.3	7.4

a Dioxane(+) indicates solvent protonated with 10 mol equiv HCl.

### Open Form: Competition between Bridge Et and Excimer Formation

The fluorescence studies above are consistent with the presence of a charge separation pathway in open **PDI-AnEt**_**2**_**-PDI**. To confirm the feasibility of this process, we calculated the thermodynamic driving force for photoexcited charge separation to form PDI^•–^AnEt_2_^•+^-PDI using the Rehm–Weller equation^41^ (see the Supporting Information for details). The resulting free energies for charge separation are summarized in Table 2 and indicate that Bridge ET (Δ*G*_CS_) is thermodynamically feasible in open **PDI-AnEt**_**2**_**-PDI** in all three of the solvent conditions. Moreover, we note that charge separation (Δ*G*_CS_) is more negative with increasing solvent polarity, owing to a more stabilized charge-separated state in the more polar solvents.^42,43^

**Table 2:** For Open **PDI-AnEt**_**2**_**-PDI**, the Hypothetical Free Energies for Charge Separation Δ*G*_CS_ and Charge Recombination Δ*G*_CR_, along with Lifetimes for the Various Processes from Global Analysis of TA Data

	bridge ET PDI^•–^AnEt_2_^•+^-PDI					
solvent	Δ*G*_CS_ (eV)	Δ**G**_CR_ (eV)	τ_CS_ (ps)	τ_CR_ (ps)	τ_PDI1*_ (ns)	τ_excimer_ (ns)
toluene	–0.79	–1.54	0.62	77	4.7	--
dioxane	–0.65	–1.71	0.47	18	5.1	--
chloroform	–1.05	–1.28	<0.20	20	3.1	>20

**Figure 3:**
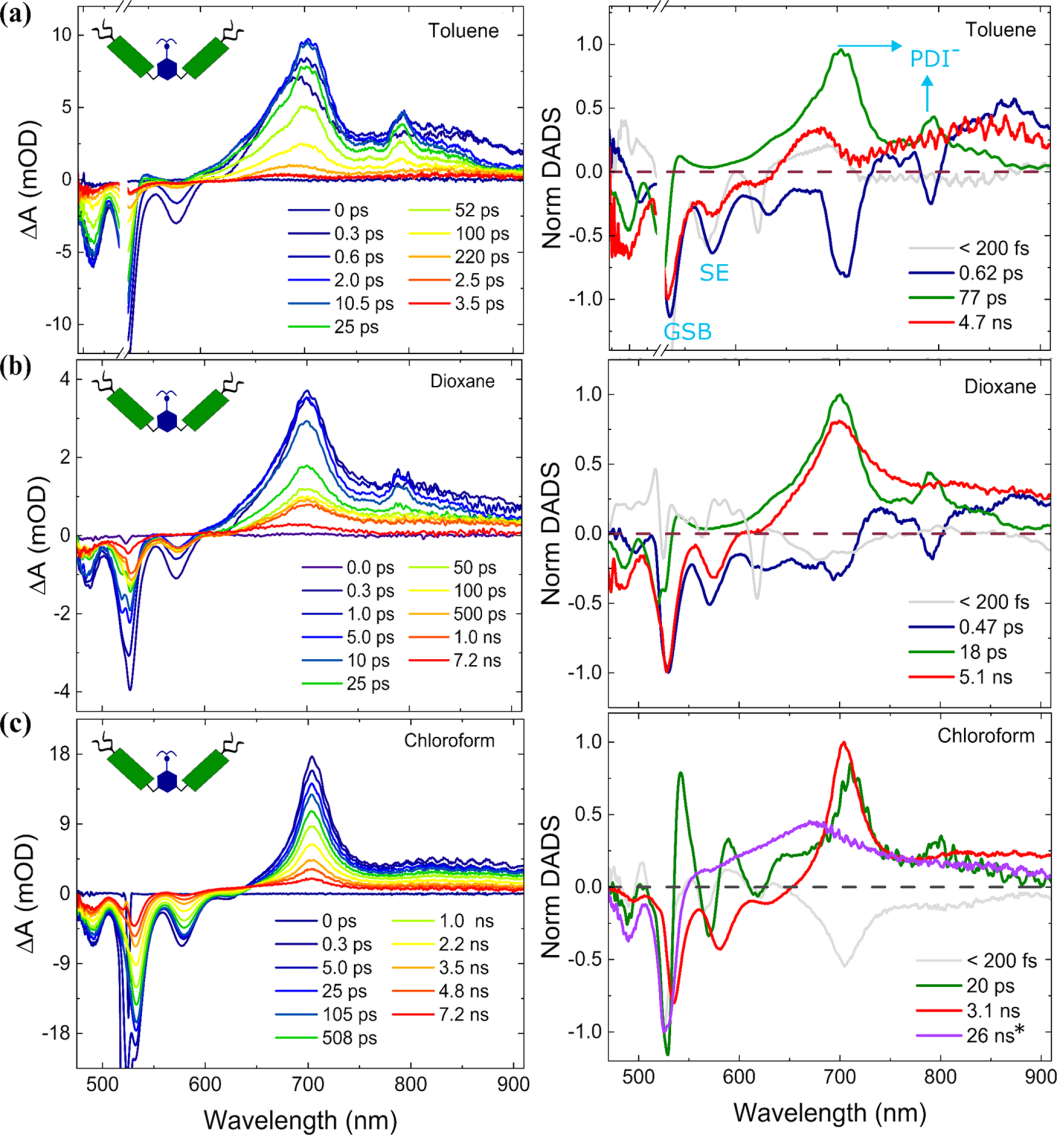
TA measurement of PDI-AnEt_2_-PDI in open form (λ_ex_ = 530 nm) in (a) toluene, (b) dioxane, and (c) chloroform. TA spectra at selected time delays are shown on the left, with the corresponding DADS from global analysis on the right. The open form exhibits photoinduced charge separation to **PDI^•–^-AnEt_2_^•+^-PDI** in all solvents (green trace in DADS). In chloroform, the PDI excimer is also formed (purple trace in DADS). *Note that the 26 ns fit for chloroform is beyond the measurement window of 8 ns. See the text for further details.

We carried out transient absorption (TA) experiments to confirm the presence of photoinduced charge separation and to measure the charge transfer dynamics and pathways of **PDI-AnEt**_**2**_**-PDI**. For open **PDI-AnEt**_**2**_**-PDI**, the samples were photoexcited at λ_ex_ = 530 nm to selectively excite PDI. Figure 3 shows TA spectral traces at selected time delays after photoexcitation, along with DADS from global analysis (see the Experimental Details section) for open **PDI-AnEt**_**2**_**-PDI** in toluene, dioxane, and chloroform. The spectra show ground-state bleach (GSB) and stimulated emission (SE) features similar to those of **PDI-ref** (Figure S14). However, in contrast to **PDI-ref**, TA spectra of **PDI-AnEt**_**2**_**-PDI** in toluene and dioxane (Figure 3a,b) exhibit a strong quenching of SE and a concomitant appearance of excited state absorption (ESA) peaks at 702 and 794 nm (toluene) and at 699 and 791 nm (dioxane). These two peaks are distinct from the single positive ESA at 705 nm in **PDI-ref**. In **PDI-ref**, the ESA is assigned to the PDI singlet excited state (PDI^1*^), which decays in parallel to the GSB/SE recovery in ∼4 ns (Figure S14). In contrast, the ESA observed for **PDI-AnEt**_**2**_**-PDI** in toluene and dioxane correspond well to the characteristic PDI radical anion absorption,^44−46^ confirming photoinduced charge separation with the bridging AnEt_2_ (Bridge ET). In the case of **PDI-AnEt**_**2**_**-PDI** in chloroform (Figure 3c), the SE quenching is not as strong and the ESA appears more similar to **PDI-ref**. However, at later delay times, the ESA of **PDI-AnEt**_**2**_**-PDI** in chloroform transforms from a narrow peak at 705 nm to a broad feature between 625 and 750 nm (Figure S15), suggesting there are underlying processes that are not clear from the TA spectral traces alone.

TA measurements are fit with a 4-compartment global model, yielding the associated DADS in Figure 3. The global fits for toluene and dioxane exhibit similar trends. The first component, DADS1 (light gray), decays faster than the 200 fs instrument response function (IRF) and is attributed to a coherent artifact in all cases. DADS2 (blue) shows spectral features consistent with the GSB and SE between 470–650 nm, as well as the growth of ESA features corresponding to the PDI radical anion at 700 and 790 nm (negative peaks in DADS2). DADS2 decays in 0.62 ps (DADS2_toluene_) and 0.45 ps (DADS2_dioxane_), and is assigned to photoinduced charge separation via Bridge ET. DADS3 (green) shows positive ESA features corresponding to PDI radical anion and no SE contribution. These decay in 77 ps (DADS3_toluene_) and 18 ps (DADS3_dioxane_), and are assigned to the charge recombination from Bridge ET. The last DADS4 (red trace) exhibits a line shape very similar to that of PDI^1*^ in **PDI-ref** (Figure S14), and decays in ∼5 ns in both toluene and dioxane. The 5 ns decay is also consistent with the lifetime of PDI^1*^. The appearance of this feature suggests that charge separation and singlet excited state decay compete in toluene and dioxane.

In the global analysis of **PDI-AnEt**_**2**_**-PDI** in chloroform (Figure 3c, right), the TA spectral line shape described by DADS2_chloroform_ corresponds to the PDI anion, indicating that charge separation has already occurred within the IRF. This charge-separated state decays in 20 ps, and this is assigned as the charge recombination rate in chloroform. DADS3_chloroform_ (red) matches the TA spectra of PDI^1*^, as in DADS4_toluene_ and DADS4_dioxane_, suggesting that this is again a competitive pathway to charge separation. Lastly, DADS4_chloroform_ (violet) exhibits similar GSB features as previously described but no clear SE and an ESA that extends from 550 to 900 nm, with a peak around 675 nm. This spectral line shape matches the signature of an H-stacked PDI excimer.^37^ The global analysis assigns a long decay lifetime of 26 ns to this feature, which is beyond the 8 ns window of the TA experiment. We therefore carried out further TRPL measurements in chloroform, and global analysis of that data reveals a long-lived feature with a red-shifted emission, consistent with excimer emission (Figure S16). The photoexcited dynamics of **PDI-AnEt**_**2**_**-PDI** in chloroform therefore exhibit a competition between PDI^1*^, charge separation to **PDI^•–^-AnEt_2_^•+^-PDI**, and excimer formation. Due to numerous overlapping ESA species, the photoproducts are not as immediately clear from the TA spectral traces in chloroform versus in dioxane and toluene. However, global analysis allows us to unambiguously prove charge separation via Bridge ET as well as excimer formation.

The observed rate constant of charge separation and recombination dynamics in the open **PDI-AnEt**_**2**_**-PDI** in toluene, dioxane, and chloroform can be described in the context of Marcus theory.^47,48^ Bridge ET is observed in all three solvents, and both rates of charge separation and charge recombination show weak dependence on solvent polarity. In both cases, the mechanism is slower with a decreasing solvent polarity, but the rates do not differ strongly. This suggests that the mechanisms lie slightly in the inverted region of the Marcus parabola but possibly very close in fact to the top of the parabola, consistent with a barrierless reaction. However, despite being a barrierless reaction, there are competitive processes, namely, the observation of PDI^1*^ in all solvents and also excimer formation in the case of chloroform. Furthermore, the TA measurement in protonated dioxane (Figure S17) follows very similar spectroscopic features as observed in chloroform, including an excimeric feature at long (>5 ns) delay times. In the case of protonated dioxane, however, no charge separation is expected. Protonated dioxane also exhibits a PLQY similar to that of chloroform (Table 1), which is, furthermore, much higher than those of the other solvents. Additionally, the steady-state fluorescence and TRPL of **PDI-AnEt**_**2**_**-PDI** in dioxane (Figures 1 and S12) also suggest some excimeric contribution, although it could not be temporally resolved and was also not clearly observed in the TA experiments. The relatively high PLQY in chloroform and protonated dioxane can therefore be explained as a result of a strong thermodynamic competition among Bridge ET, excimer formation, and excited state relaxation even in the *open* solvent conditions. The fact that excimer forms in chloroform and protonated dioxane, despite the expected low binding coefficients, may point to some charge transfer character in the excimer, which is stabilized by the more polar solvent conditions.

### Folded Form: Competitive Charge Separation between Bridge ET and SBCS

In the solvent conditions that facilitate the formation of folded **PDI-AnEt**_**2**_**-PDI**, the two PDI chromophores are in close proximity, and so we also consider the possibility of symmetry-breaking charge separation (SBCS). In SBCS, the π-stacked coupling leads to charge separation between the two identical PDI chromophores. This mechanism has been previously observed in similar PDI dimers^10,18^ and is also consistent with the charge transfer character hypothesized for the excimeric state in the open form. We therefore calculated the thermodynamic free energies for two charge separation photoproducts: **PDI^•–^-AnEt_2_^•+^-PDI** and **PDI^•–^-AnEt_2_-PDI^•+^**. As shown in Table 3, the negative values for Δ*G*_CS_ indicate thermodynamically favorable conditions for both charge separation pathways. In cyclohexane, SBCS is predicted to be more favorable than Bridge ET, while the opposite is expected in acetone and DMSO.

**Table 3:** For folded **PDI-AnEt**_**2**_**-PDI**, the Hypothetical Free Energies for Charge Separation Δ*G*_CS_ and Charge Recombination Δ*G*_CR_, along with Lifetimes for the Various Processes, Were Observed from Global Analysis of TA Data

	bridge ET **PDI**^•**–**^**-AnEt**_**2**_^•**+**^**-PDI**		SBCS **PDI**^•–^**-AnEt**_**2**_**-PDI**^•**+**^		bridge ET	SBCS	
solvent	Δ**G**_CS_ (eV)	Δ*G*_CR_ (eV)	Δ**G**_CS_ (eV)	Δ**G**_CR_ (eV)	τ_CR_ (ps)	τ_CR_ (ps)	τ_PDI1*_ (ns)
cyclohexane	–0.42	–1.97	–1.30	–1.10	8.9	143	4.4
acetone	–1.20	–1.17	–0.37	–2.0	3.5	115	4.2
DMSO	–1.20	–1.12	–0.27	–2.05	4.0	148	1.4

**Figure 4:**
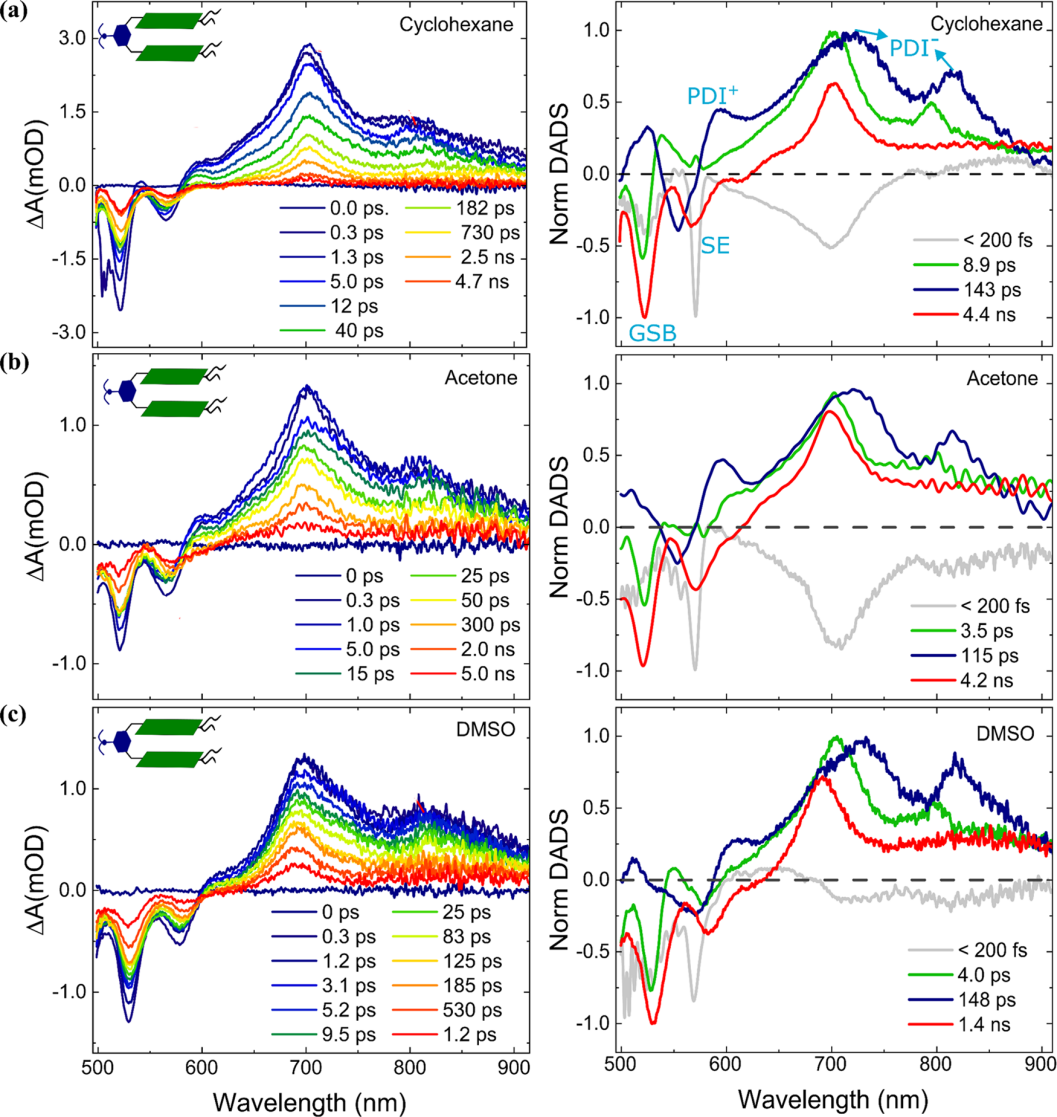
TA measurement of **PDI-AnEt_2_-PDI** in folded form (λ_ex_ = 490 nm) in (a) DMSO, (b) acetone, and (c) cyclohexane. TA spectra at selected time delays are shown on the left with the corresponding DADS from global analysis on the right. The folded form shows two charge separation photoproducts: **PDI**^•–^**-AnEt**_**2**_^•**+**^**-PDI** (green trace in DADS) and **PDI**^•–^**-AnEt**_**2**_**-PDI**^•**+**^ (blue trace in DADS).

Figure 4 shows TA spectral traces for folded **PDI-AnEt**_**2**_**-PDI** measured in cyclohexane, acetone, and DMSO. The TA data are measured with λ_ex_ = 490 nm to maximize signal-to-noise and suppress scattering, although similar dynamics are seen with λ_ex_ = 530 nm (Figure S18). All of the solvents display similar features, with GSB at 530 nm and SE at 580 nm. The ESA features occur over a broad spectral range between 600 and 900 nm, with peaks around 700 and 800 nm consistent with the PDI anion peak. However, we do not see significant quenching of the SE peak except at very early times. Additionally, the ESA features are broader than what was observed in the open form. A new ESA peak at around 590 nm appears in these spectra. The spectral traces also indicate that the overall photophysical dynamics decay much faster in DMSO, where the TA spectra at 1.2 ps exhibit a similar ΔA as that at 4.7–5.0 ns in acetone and cyclohexane. This is in agreement with the shorter PL lifetime and lower PLQY in DMSO (Table 1).

The TA data for the folded form are fit using a 4-compartment global model, yielding the DADS shown on the right side of Figure 4. The global fits exhibit a similar trend in all three solvents that stabilize the folded form. As mentioned above, the first component, DADS1 (light gray), is attributed to the coherent artifact due to the IRF. After the IRF, the TA spectra exhibit a line shape described by DADS2 (green) spectra, which has the spectral features of GSB and ESA with peaks at 705 and 803 nm and no SE contribution, matching the signature of PDI radical anion. The decay of DADS2 is assigned to the charge recombination process of **PDI**^•**–**^**-AnEt**_**2**_^•**+**^**-PDI** and is fit to rates of 4.0 ps (DADS2_DMSO_), 3.5 ps (DADS2_acetone_), and 8.9 ps (DADS2_cyclohexane_). This trend indicates a weak dependence of charge recombination on solvent polarity, indicating the Bridge ET recombination reaction to be barrierless or slightly in the inverted regime of the Marcus parabola. DADS3 (blue) exhibits similar ESA features at 705 and 805 nm, as well as a peak at 590 nm. While a peak at this spectral position could indicate excimer absorption, an excimer ESA would be expected to live for nanoseconds, as observed in the open chloroform condition (Figure 3c). Previous reports indicate that PDI cation exhibits an ESA at 590 nm.^44,49^ The coexistence of PDI radical cation and radical anion peaks in folded **PDI-AnEt**_**2**_**-PDI** form DADS3 with identical decay dynamics suggests that the charge separation occurs between two PDI chromophores through SBCS and the time constant associated with DADS3 is assigned to the charge recombination of **PDI**^•–^**-AnEt**_**2**_**-PDI**^•**+**^. This compartment decays in 148 ps (DADS3_DMSO_), 115 ps (DADS3_acetone_), and 143 ps (DADS3_cyclohexane_). The lack of any clear solvent polarity dependence for recombination of the SB state indicates a barrierless reaction. DADS4 (red) exhibits line shape features that reproduce the PDI excited state (PDI^1*^) decay spectra. This decays in ∼5 ns in acetone and cyclohexane and with a much faster rate of 1.4 ns in DMSO. Photoexcited folded **PDI-AnEt**_**2**_**-PDI** thus exhibits a competition between two different charge separation pathways and PDI excited state decay. There is also no clear evidence of excimer formation in the folded form, despite the strong H-stacking seen in the steady-state absorption (Figure 1).

### Flexible Conformation of **PDI-AnEt**_**2**_**-PDI** Offers Insight into Mediating Competitive Pathways

**Figure 5:**
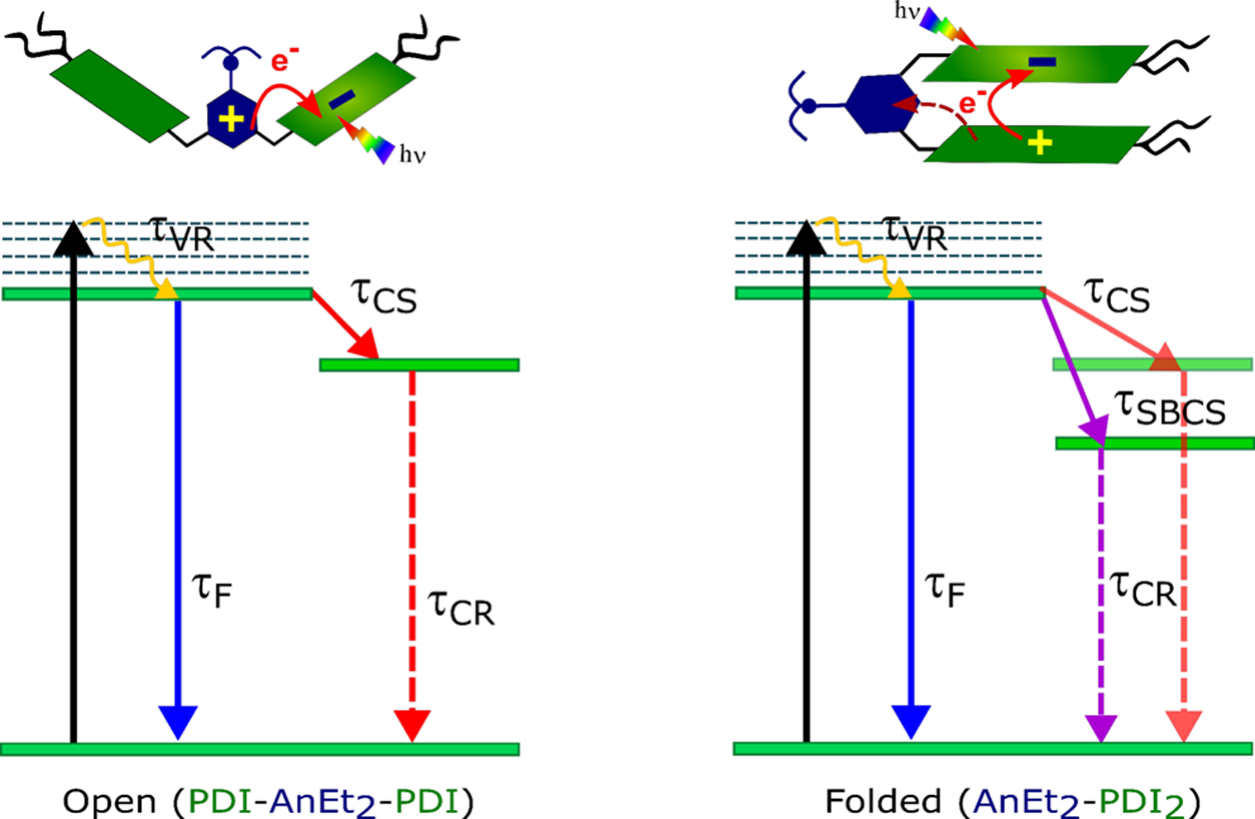
Summary of photoexcited pathways in **PDI-AnEt_2_-PDI** (energies not to scale): In the open form, photoinduced electron transfer from the bridging donor yields **PDI^•–^-AnEt_2_^•+^-PDI**. In the folded form, two different charge separation pathways compete to form both **PDI^•–^-AnEt_2_^•+^-PDI** and also **PDI^•–^-AnEt_2_-PDI^•+^** via symmetry-breaking charge separation.

The photophysical characterizations described above indicate some notable differences between open and folded **PDI-AnEt**_**2**_**-PDI**, summarized in Figure 5. The open form exhibits competition among Bridge ET, excimer formation, and PDI excited state decay. The folded form exhibits two competitive charge separation pathways, Bridge ET and SBCS, which furthermore compete with PDI excited state decay and presents no evidence of excimer formation.

In the open form, both the forward and reverse charge separation pathways could be spectroscopically resolved in TA. In the folded form, however, the forward charge separation rate could not be resolved for either of the two observed pathways. Attempts to globally analyze the data with more compartments indicated overfitting. Furthermore, the charge recombination of the Bridge ET (**PDI**^•**–**^**-AnEt**_**2**_^•**+**^**-PDI**) is also faster in the folded form (4–9 vs 20–80 ps in the open form). This suggests that the forward charge separation for Bridge ET in the folded form is faster than that in the *open* form, occurring within the IRF. The forward rate of SBCS is also not resolved. This may be occurring also within the IRF, or possibly competitively with the charge recombination from the Bridge ET. The folded form is geometrically more constrained than the open form, which may facilitate a more efficient charge separation as well as a faster charge recombination via Bridge ET.

Open **PDI-AnEt**_**2**_**-PDI** in chloroform and protonated dioxane yield evidence of dynamic excimer formation, in addition to Bridge ET. This is somewhat unexpected for the open conformation. It is possible that the charge-separated intermediate facilitates folding and excimer formation. Previous work on a structurally similar PDI folda-dimer has shown that redox events can precipitate structural changes.^34^ This suggests that the flexibility of **PDI-AnEt**_**2**_**-PDI** is also playing a dynamic role in mediating the photophysics, a finding that could be exploited in the future for designed functionality.

Folded **PDI-AnEt**_**2**_**-PDI** exhibits a competition between two charge separation pathways—the Bridge ET seen in the open form and also SBCS between the two PDI chromophores. Both processes are predicted to be thermodynamically favorable, and our data analysis could not reasonably resolve significant differences in the branching ratio or charge separation rates. The presence of the two pathways in all solvents suggests similar ion-pair energies for both charge-separated photoproducts. We propose that the competitive balance between these pathways is mediated by the conformational flexibility of PDI π-stacking. Unlike other similar systems,^16^ the PDI interaction in **PDI-AnEt**_**2**_**-PDI** is mediated by only one flexible anchor, which can allow for the molecules to explore a large space of aggregation geometries and electronic couplings.

The folded form was furthermore expected to undergo excimer formation due to the H-stacking evidence in the steady-state absorption, yet no excimeric ESA is observed in the DADS. Referring back to the TRPL measurements (Figures S12 and 13), we note the absence of any significant excimer emission in any of the folded form solvents. The lack of any excimeric contribution in the TRPL or TA of folded **PDI-AnEt**_**2**_**-PDI** suggests that the SBCS pathway dominates over excimer formation. The solvent polarity-dependent Stokes shift in the folded form PL spectra also supports a strong charge transfer character, which would facilitate SBCS. It is possible that excimer formation precedes the SBCS, for example, as from literature reports which indicate that SBCS can be mediated by an excimeric intermediate.^16,50^ But this cannot be confirmed or refuted from the present data. Other work has also shown the reverse, wherein states with strong CT character will first exhibit signatures of charge separation, followed by formation of the excimer,^51,52^ but we see no evidence of this in the folded form.

Furthermore, while the charge recombination of Bridge ET in folded **PDI-AnEt**_**2**_**-PDI** is slightly dependent on solvent polarity, the recombination from the SBCS pathway does not show any clear trend with respect to solvent polarity. The charge recombination within 150 ps is faster than observed in similar systems.^16,53^ Typically, SBCS is not expected to occur in nonpolar environments, due to destabilization of the CT state. However, the mechanism can still proceed when the geometry of the system is constrained in such a way as to promote CT coupling.

We also note that the TRPL decay lifetimes match closely with those of the DADS associated with PDI^1*^. PDI has a near-unity quantum yield,^26^ while the other pathways (charge separation, excimer formation) will quench PDI fluorescence. Therefore, the TRPL measurements are primarily reporting on the competitive PDI^1*^ pathway, which is observed in all of the solvents, regardless of conformation. We also note the lack of triplet formation in this work, indicating that in our **PDI-AnEt**_**2**_**-PDI** system, the excited state mixing lies predominantly between the singlet Frenkel exciton and charge transfer conditions.^9^

This is consistent with the conclusion that the excited potential energy surface of **PDI-AnEt**_**2**_**-PDI** exhibits significant mixing between (quasi)diabatic states with the photoexcited population competing between various thermodynamically favorable pathways. This condition is expected in folded **PDI-AnEt**_**2**_**-PDI**, due to the π-stacked dimer in this condition. Our measurements show that even for open **PDI-AnEt**_**2**_**-PDI**, the Bridge ET pathway competes with other processes, facilitated by the conformational flexibility of the molecular system. In the context of understanding these materials for applications, such competition presents a potentially significant barrier. However, we also unexpectedly found preferential SBCS over excimer formation in the folded form. Excimers are typically dark, undesirable traps, whereas SBCS can provide a functional pathway for producing charge carriers for photovoltaics. These results can therefore provide insight into how conformationally flexible folda-dimer systems can be designed and built to tune competitive photophysical pathways.

## Conclusions

We have synthesized a conformationally flexible molecular donor–acceptor–donor system **PDI-AnEt_2_-PDI** which adopts either an open or folded conformation depending on the solvent environment. Spectroscopic characterization shows that this system exhibits multiple photophysical pathways, and the competition between these processes is mediated by an interplay of solvent environment and molecular conformation and flexibility. In the open form, we see evidence of dynamic conformational change depending on the solvent environment. This gives insight into the development and study of molecular actuator systems, particularly in combination with light-activation. In the folded form, we observe a preference for SBCS over excimer formation, results that can inform future development of molecular systems with preferential symmetry-breaking charge separation character.

## Supporting Information Available

Synthetic scheme, procedures, and characterization; solvent-dependent binding energies for PDI π-stacking; concentration-dependent steady-state measurements; cyclic voltammetry; details regarding calculating free energies for charge separation; TRPL; and additional TA data (PDF)
