# Supplementary material for: Competitive Charge Separation Pathways in a Flexible Molecular Folda-Dimer
Source: J Phys Chem B. 2024 Feb 10;128(7):1760–70. doi: 10.1021/acs.jpcb.3c07134 (PMC10895663; doi:10.1021/acs.jpcb.3c07134)
Supplement: Supplementary file 1 — jp3c07134_si_001.pdf [file jp3c07134_si_001.pdf]

Supplementary Information

for

Competitive Charge Separation Pathways in a Flexible Molecular Folda-dimer

Kalyani Thakur[1], Saptarshi Datta[2], Paul W.M. Blom[1], Debangshu Chaudhuri[2*], Charusheela Ramanan[1,3,*]

[1] Max Planck Institute for Polymer Research, Ackermannweg 10, Mainz 55128, Germany

[2] Department of Chemical Sciences, Indian Institute of Science Education and Research (IISER) Kolkata, Mohanpur 741246, India

[3] Department of Physics and Astronomy, Vrije Universiteit Amsterdam, De Boelelaan 1081, 1081HV, Amsterdam, Netherlands

*corresponding authors: c.ramanan@vu.nl, dchaudhuri@iiserkol.ac.in

Synthesis and Characterisation 2
Synthetic Scheme 2
Synthetic Procedures 3
Solvent Dependent Binding Energies for PDI π-stacking 11
Concentration Dependent Steady-State Absorption and Photoluminescence 12
Calculating Free Energies for Charge Separation 13
Redox Potentials from Cyclic Voltammetry 13
Molecular Geometries and Energies 14
Thermodynamic Driving Force using Rehm-Weller expression 14
Figure S12, S13 – Time-Resolved Photoluminsecence (TRPL) 16
Figure S14 – TA of PDI Ref 18
Figure S15 – Comparing TA lineshapes of PDI-AnEt$_2$-PDI in chloroform and PDI-Ref 19
Figure S16 – TRPL of PDI-AnEt$_2$-PDI in chloroform, 50 ns measurement window 20
Figure S17 – TA of PDI-AnEt$_2$-PDI in protonated dioxane (open form) 21
Figure S18 – Comparing TA of PDI-AnEt$_2$-PDI in acetone (folded form) at λ_{ex} = 490 and λ_{ex} = 530 nm 22

Synthesis and Characterisation

Synthetic Scheme

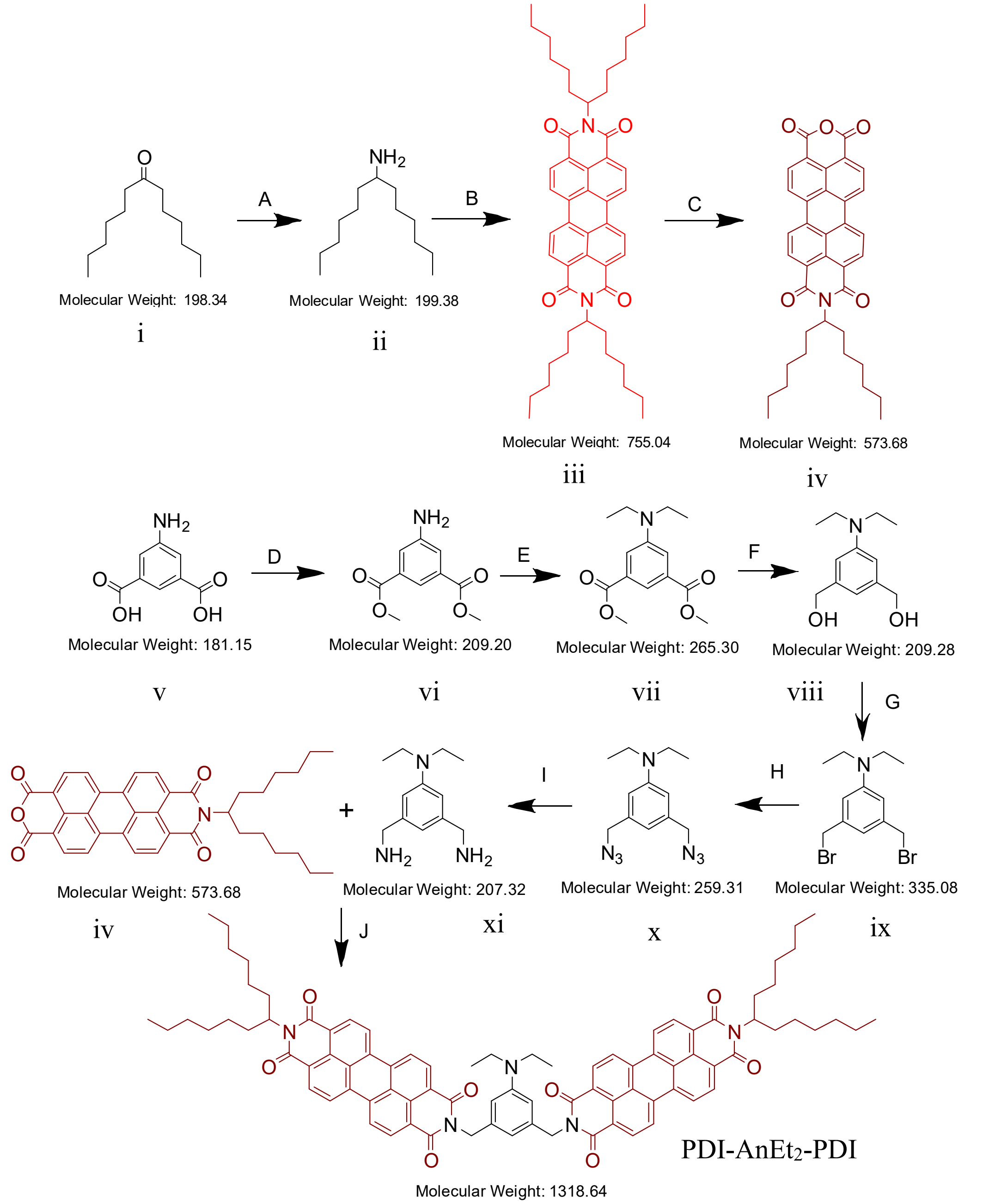

A) NH_4OAc, $NaBH_3CN$, dry MeOH ,48 hours in room temperature; B) PBA (Molecular Weight: 392.32), Imidazole, 3 hours in 130 ^{0}C; C) 1. KOH / t-butanol, reflux 100 ^{0}C; AcOH; HCl; K_2CO_3 solution, reflux 100 ^{0}C; Et_3N; HCl; D) $SOCl_2$ / MeOH, 8 hrs, inroom temperature; E) Na_2HPO_4 / dry DMF, Iodoethane,65 ^{0}C, 36 hrs; F) $LiAlH_4$ / dry THF, 12 hrs in 0 ^{0}C to room temperature; G) PBr_3 / dry ether, 8 hrs, room temperature; H) NaN_3 / dry DMF, 12 hrs at 90 ^{0}C; I) H_2 / 10% Pd-C,dry ethyl acetate, 12 hrs in room temperature; J) imidazole, 130 ^{0}C

Synthetic Procedures

Reductive amination to prepare ii

The synthesis was carried out following the procedure described in [1]. In a 100 ml round-bottom flask, ammonium acetate (11 g, 142.7 mmol) was dissolved in 40 ml dry methanol, and then 7-tridecanone (i) (2.5 g, 12.6 mmol) was added to this mixture. After 20 minutes, sodium cyanoborohydride (1 g, 15.9 mmol) was added, and the mixture was stirred at room temperature for 48 hours. The ninhydrin test confirmed the completion of the reaction. The reaction was quenched by dropwise addition of 3 ml of concentrated hydrochloric acid, precipitating a white crystalline solid. The methanol was removed via a rotary evaporator, and the white solid was subsequently dissolved in 200 ml aqueous KOH solution of pH 10. This solution was taken in a separatory funnel and washed with dichloromethane (60 ml x 3). The organic fraction was dried over anhydrous Na_2SO_4 and then concentrated by a rotary evaporator to obtain the pale-yellow liquid of ii. (Yield 91%)

[1] Samanta S. and Chaudhuri D., *The Journal of Physical Chemistry Letters* 2017 **8** (14), 3427-3432

Perylene diimide(iii) preparation:

The synthesis was carried out following the procedure described in [1]. Imidazole (6 g) was taken in a two-neck round-bottom flask and heated at 130 ^{0}C under argon atmosphere until completely melted. Next, perylene- 3, 4, 9, 10 - tetracarboxylic dianhydride (1.4 g, 3.57 mmol) was added to the hot liquid imidazole followed by freshly prepared amine ii (2.3 g, 11.54 mmol). This reaction mixture was refluxed at 130 ^{0}C under argon atmosphere for 3 hours with continuous stirring. Next, the heating was stopped, and the reaction was left to cool to room temperature. During the cooling process, 100 ml n-butanol was added to the reaction mixture to avoid solidification of the reaction mixture. To this reaction mixture, 200 ml 3(M) HCl solution was added and stirred overnight at room temperature and under argon. The dark red precipitate was filtered, washed with distilled water, and dried to yield Perylene diimide, iii (red solid, yielding 92%).

Perylene monoimide(iv) preparation:

A 250 ml round-bottom flask, charged with prepared perylene diimide, iii (1 g, 1.32 mmol), was dissolved in 60 ml t-butanol. Then KOH (0.7 g, 12.5 mmol) was added to the mixture. This reaction mixture was refluxed at 100 ^{0}C under argon atmosphere for an hour with continuous stirring. Next, the reaction mixture was left to cool down and was transferred to a 500 ml round-bottom flask. 30 ml acetic acid was added to this dark red solution and was stirred for 1 hour, and after that, 200 ml 3(M) HCl solution was added and stirred overnight at room temperature and under argon. The red precipitate was filtered and washed with distilled water.

10% aqueous K2CO3 solution (5 grams in 50 ml water) was added to this red solid. This mixture was refluxed at 100 ^{0}C with continuous stirring for 1 hour. After that, the reaction mixture was cooled to room temperature and the brown precipitate was filtered and washed with distilled water. This brown solid was dissolved in triethylamine (~100 ml). 6(M) HCl solution was added dropwise to induce a reddish-brown product to precipitate. This mixture

was stirred overnight at room temperature under argon. The reddish-brown precipitate was filtered, washed with distilled water and dried to yield 30% of perylene monoamide, iv.

Esterification reaction(vi):

In a 100 ml round-bottom flask, 5-aminoisophthalic acid, v (3 g, 16.56 mmol) was dissolved in 20 ml methanol. Then thionyl chloride (10 ml, 137.85 mmol) was added dropwise, and this mixture was stirred for 8 hours at room temperature and a white crystalline solid precipitated. Methanol with thionyl chloride was removed by distillation, and the resulting white solid was dissolved in 70 ml ice cold water. This solution was transferred to a 250 ml round-bottom flask, and 100 ml 10% Na_2CO_3 solution was subsequently added. This mixture was stirred for half an hour and then was washed in a separatory funnel with dichloromethane (70 ml x 3). The organic fraction was dried over anhydrous Na_2SO_4, filtered, and then the solvent was removed using a rotary evaporator to obtain dimethyl-5-amino isophthalate, vi (Yield 84%).

N, N-diethylation reaction(vii):

In a 50 ml round-bottom flask, dimethyl-5-aminoisophthalate, vi (2.9 g, 13.86 mmol) was dissolved in 25 ml dry dimethylformamide, and then disodium hydrogen phosphate (12 g, 84.53 mmol) was added into this solution. Next, iodoethane (8 ml, 100 mmol) was added to this solution, and this mixture was refluxed at 65 ^{0}C under argon atmosphere for 36 hours with continuous stirring. After 36 hours, the reaction mixture was diluted with 150 ml of water and extracted with ethyl acetate (50 ml x 3). The organic fraction was washed with brine solution, dried over sodium sulfate, filtered, and concentrated. Next, 15 ml n-pentane was added to this yellow solid and the mixture was sonicated and then filtered. The filtrate was concentrated, and a pale-yellow solid was obtained. Starting from 2.9 grams of dimethyl-5-aminoisophthalate, 3.19 grams of dimethyl-5-(diethylamino)isophthalate, vii (87% yield) was obtained.

Reduction of vii:

Dimethyl-5-(diethylamino)isophthalate, vii (1g, 3.77 mmol) was dissolved in 20 ml dry tetrahydrofuran. The flask was cooled to -10 ^{0}C using an ice/acetone bath, and $LiAlH_4$ (0.36 g, 9.4 mmol) was added dropwise, and this mixture was left overnight to warm to room temperature. In an ice-water bath, the reaction was quenched with a minimum volume of ice-cold water and 25 ml of ethyl acetate. This mixture was stirred for half an hour. Next, this mixture was filtered, and the filtrate was concentrated to yield a pale yellow waxy solid of (5-(diethylamino)-1,3-phenylene)dimethanol, viii (76% yield).

Bromination of viii:

(5-(diethylamino)-1, 3-phenylene) dimethanol, viii (0.2g, 0.956 mmol) was dissolved in 20 ml dry ether. To this, a solution of PBr_3 (0.2 ml, 1.434 mmol) in 10 ml dry ether was added at room temperature. This mixture was kept for 8 hours with continuous stirring. After that, this reaction mixture was neutralised by dropwise addition of 10% aqueous Na_2CO_3 solution until the effervescence of CO_2 stopped. The reaction mixture was washed with dichloromethane (50

ml x 3). The organic fraction was washed with brine solution, dried over sodium sulfate, filtered, and concentrated to obtain 3,5-bis(bromomethyl)-N, N-diethylaniline, ix (81% yield).

Preparation of azide(x):

3,5-bis(bromomethyl)-N, N-diethylaniline, ix (0.2g, 0.597 mmol) was dissolved in 20 ml dry dimethylformamide. Sodium azide (0.16 g, 2.38 mmol) was added, and this mixture was kept for 12 hours at 90 ^{0}C with continuous stirring. After that, this reaction mixture was diluted by adding 100 ml water and stirred overnight. The reaction mixture was washed with ethyl acetate (50 ml x 3), and the organic fraction was washed with brine solution, dried over sodium sulfate, filtered, and concentrated to obtain 3,5-bis(azidomethyl)-N,N-diethylaniline, x (84% yield).

Preparation of amine: -

3,5-bis(azidomethyl)-N,N-diethylaniline, x (0.13 g, 0.502 mmol) was dissolved in 20 ml dry ethyl acetate, and subsequently, 10% Pd-C (0.05 g, 0.47 mmol) was added. This mixture was kept for 12 hours at room temperature under the pressure of hydrogen gas with continuous stirring. After that, this reaction mixture was filtered by Whatman 42 filter paper to remove charcoal and was concentrated by rotary evaporator to yield (5-(diethylamino)-1,3-phenylene)dimethanamine, xi (87% yield).

Imide formation (final step): -

Imidazole (6 g) was heated at 130 ^{0}C under argon atmosphere until it was melted completely. Next, perylene monoamide, iv (0.63 g, 1.1 mmol) was added to the hot liquid imidazole followed by freshly prepared (5-(diethylamino)-1,3-phenylene) dimethanamine, xi (0.09 g, 0.434 mmol). This reaction mixture was refluxed at 130 ^{0}C under argon atmosphere for 8 hours with continuous stirring. Next, the heating was stopped, and at 110 ^{0}C, 100 ml n-butanol was added to the reaction mixture, and it was cooled down to room temperature. To this reaction mixture, 200 ml 3 M HCl solution was added and stirred overnight. The dark red precipitate was filtered, washed with distilled water, and dried to obtain PDI-AnEt$_2$-PDI as the final product (red solid, 52% yield).

Characterisation

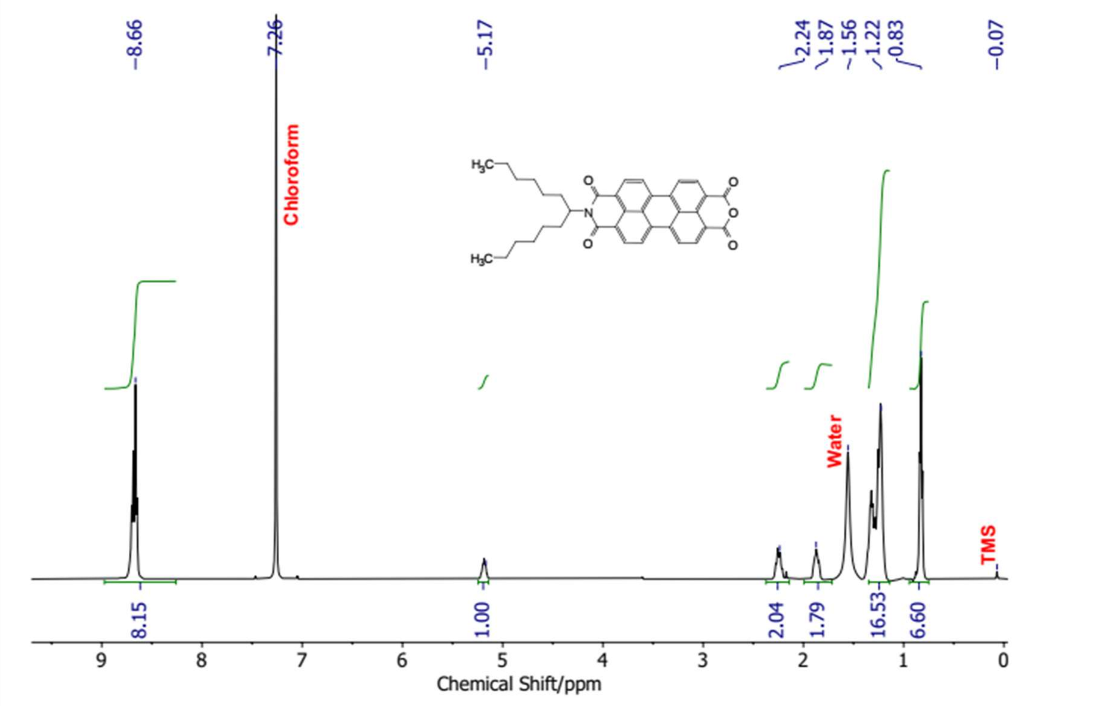

Figure S1. ^{1}H NMR of ***Perylene monoimide***

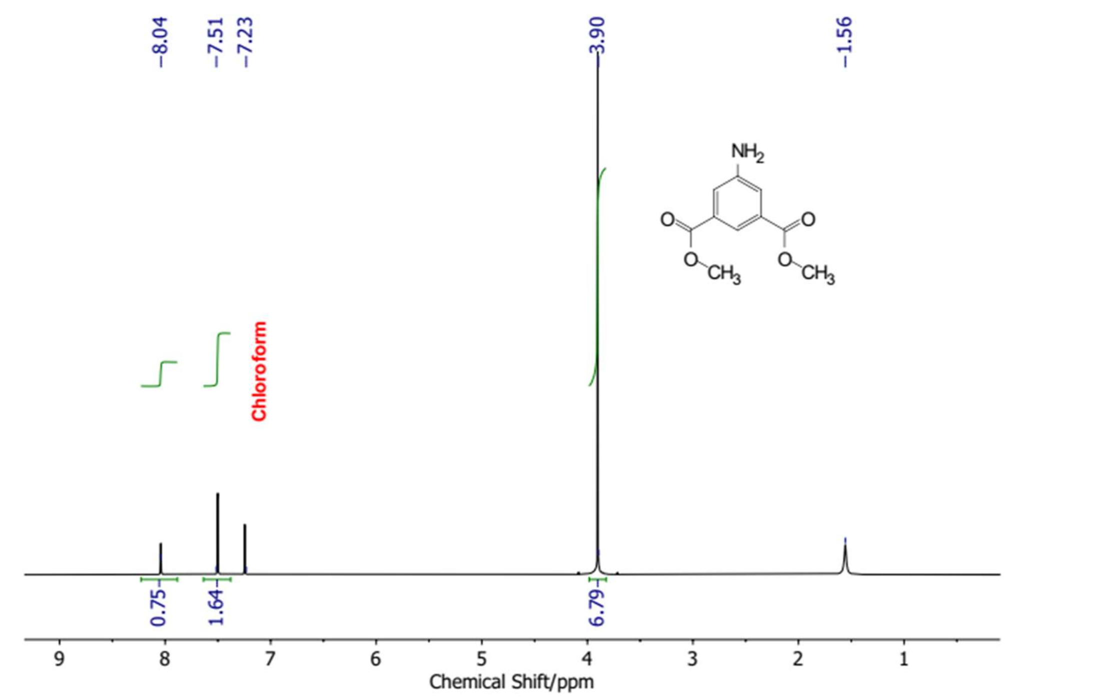

Figure S2. 1H NMR of dimethyl-5-aminoisophthalate

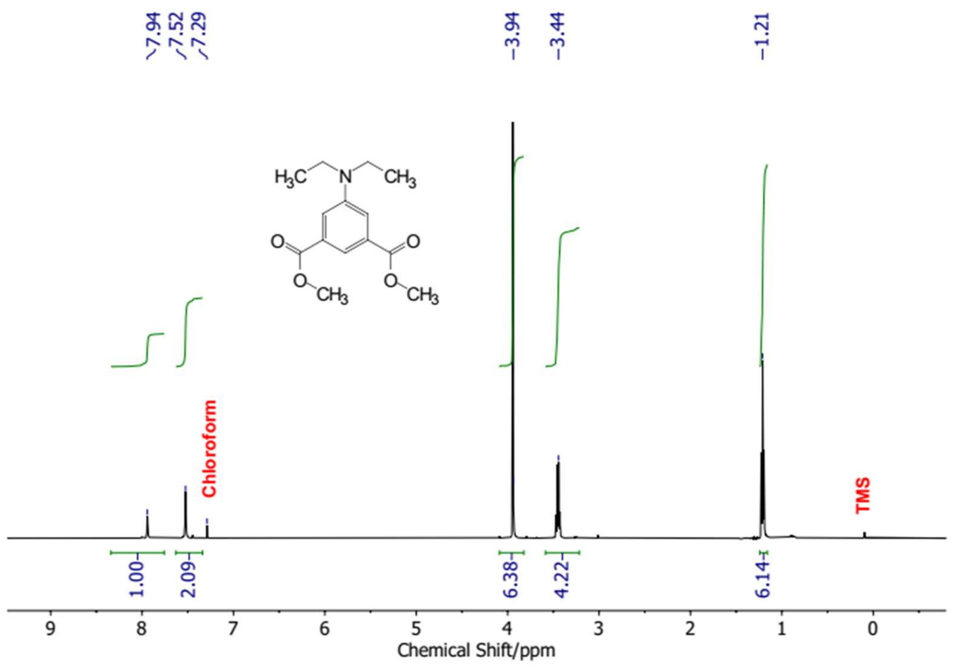

Figure S3. ^{1}H NMR of dimethyl-5-(diethylamino)isophthalate

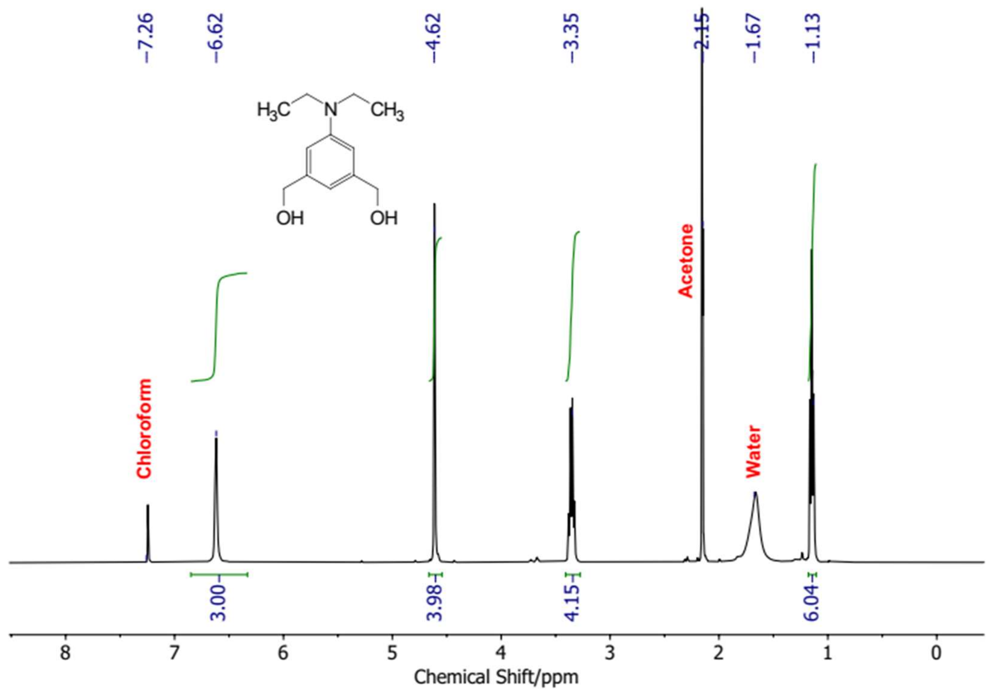

Figure S4. ^{1}H NMR of (5-(diethylamino)-1,3-phenylene)dimethanol

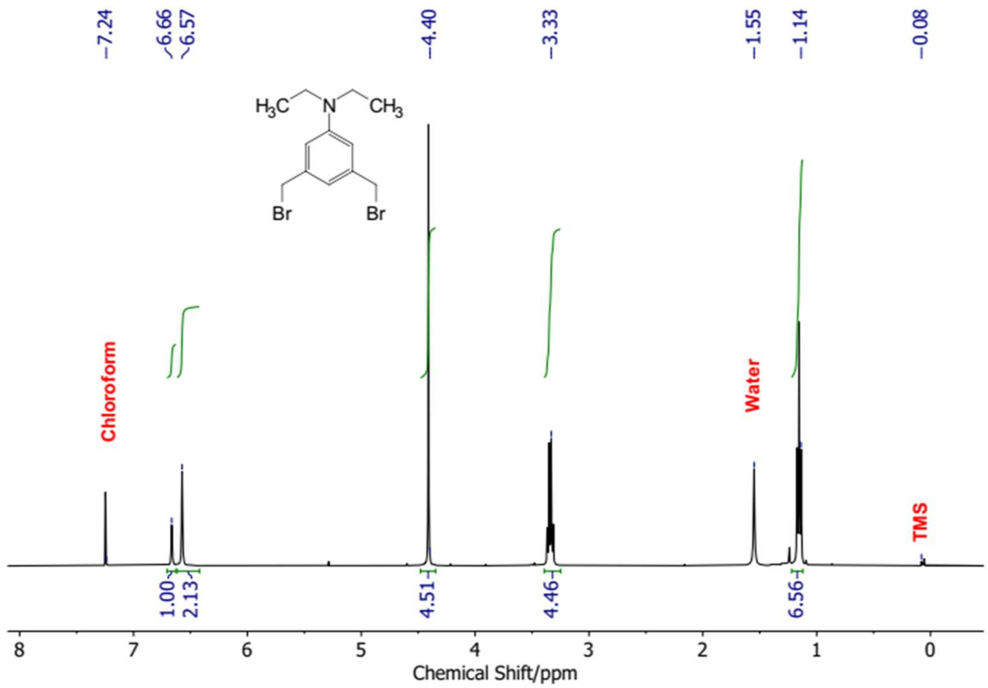

Figure S6. ^{1}H NMR of 3,5-bis(bromomethyl)-N,N-diethylaniline

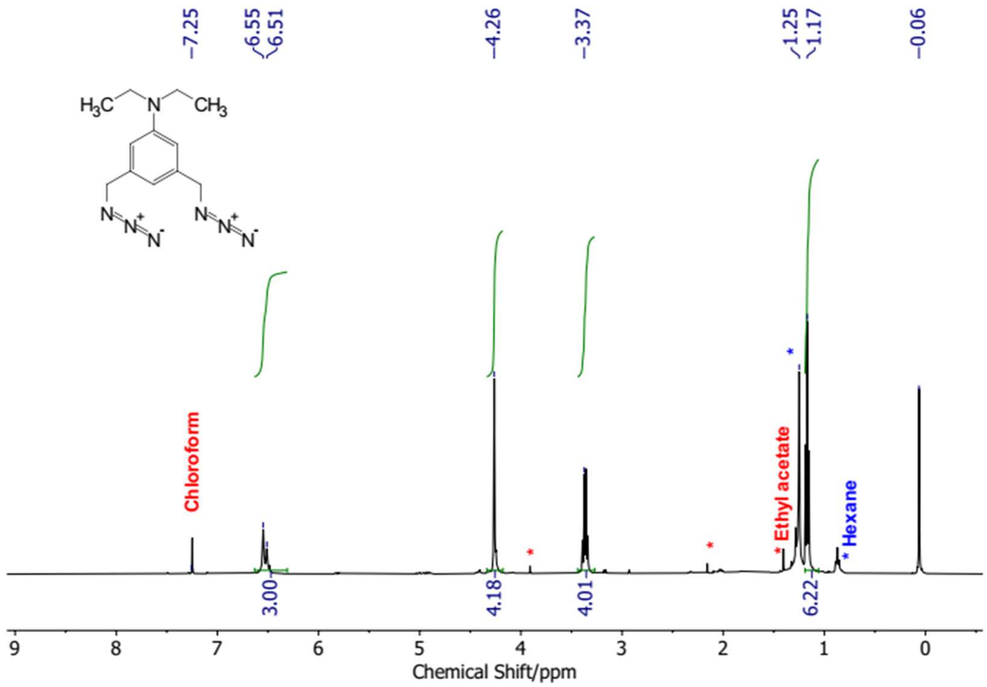

Figure S5. ^{1}H NMR of 3,5-bis(azidomethyl)-N,N-diethylaniline

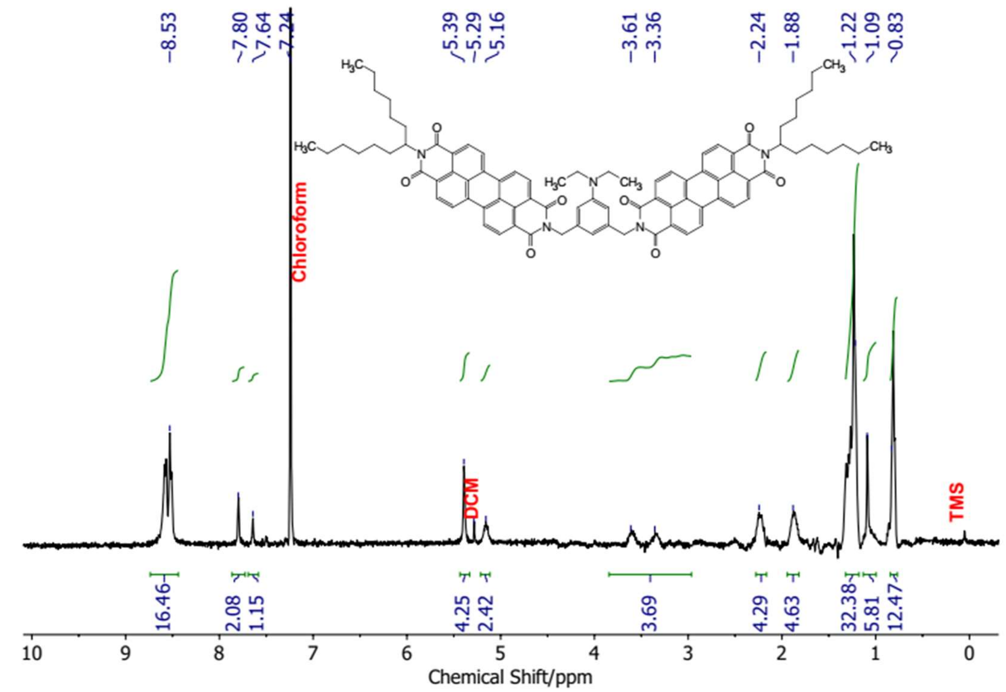

Figure S8. 1H NMR of PDI-AnEt2-PDI

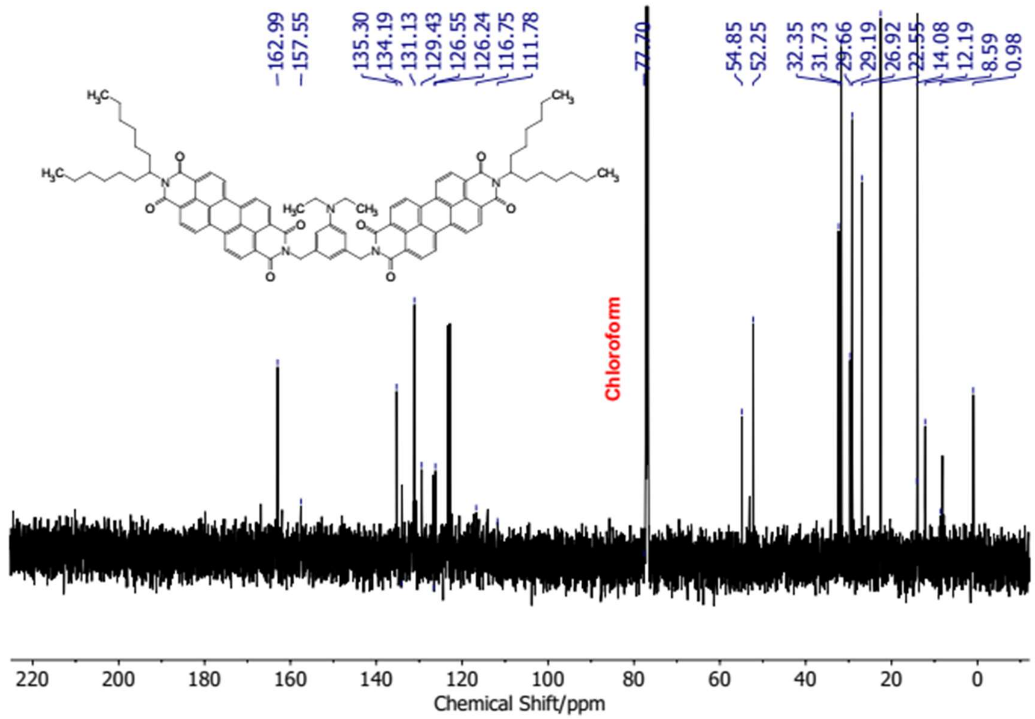

Figure S7. ^{13}C NMR of PDI-AnEt2-PDI

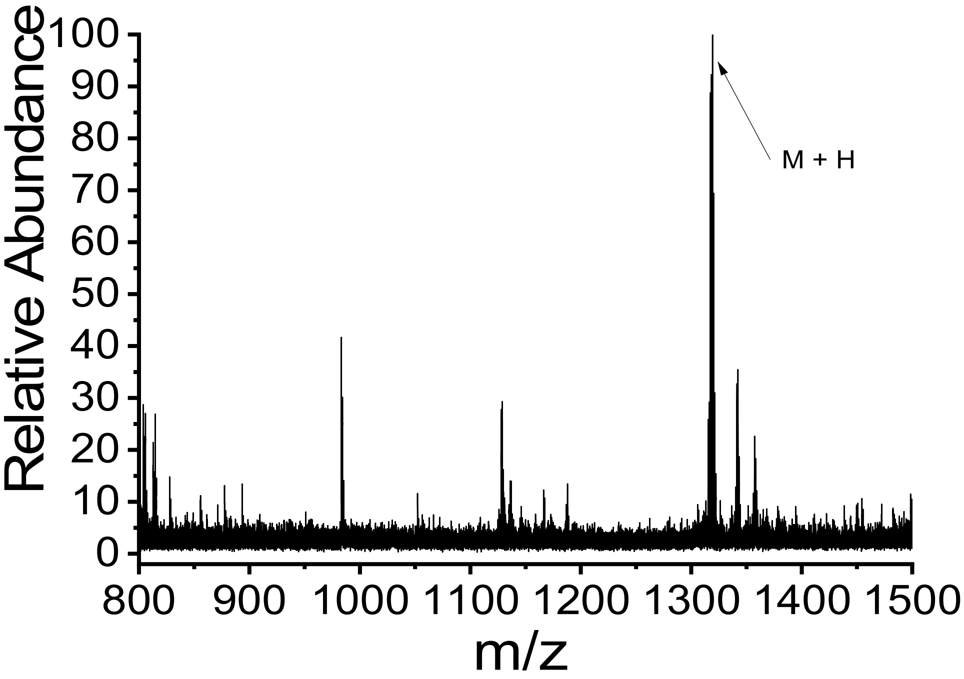

Figure S9 HRMS of PDI-AnEt$_2$-PDI

Solvent Dependent Binding Energies for PDI π-stacking

Table S1. The binding energy of PDI is due to π-stacking in different solvents, taken from literature.[1,2]

Solvent	The binding energy of PBI due to π-stacking # ($\Delta G°$/kJMol^{-1})
Chloroform	-15
1,4-Dioxane	-15
Toluene	-15
DMSO*	-17
Acetone	-20
Cyclohexane	-30

Assuming that the side chains do not contribute significantly to the intermolecular interactions

*Molecules with single PBI units never undergo aggregation in DMSO (we have also observed this), whereas di-chromophoric PBI foldemeric systems (similar to A-D-A) have been seen to give H-aggregated spectra. In DMSO, we found no change in the extent of aggregation in dilution for such systems (Figure S10), indicating intra-molecular folding only and no extended intermolecular-aggregation. These H-aggregates in DMSO do not found to have excimeric emission either. Here in the table, the binding energy in DMSO has been reported using mono-chromophoric PBI compounds.

(1) Chen, Z.; Fimmel, B.; Würthner, F. Solvent and Substituent Effects on Aggregation Constants of Perylene Bisimide π-Stacks – a Linear Free Energy Relationship Analysis. *Org. Biomol. Chem.* **2012**, *10* (30), 5845–5855. https://doi.org/10.1039/C2OB07131B.
(2) Würthner, F. Solvent Effects in Supramolecular Chemistry: Linear Free Energy Relationships for Common Intermolecular Interactions. *J. Org. Chem.* **2022**, *87* (3), 1602–1615. https://doi.org/10.1021/acs.joc.1c00625.

Concentration Dependent Steady-State Absorption and Photoluminescence

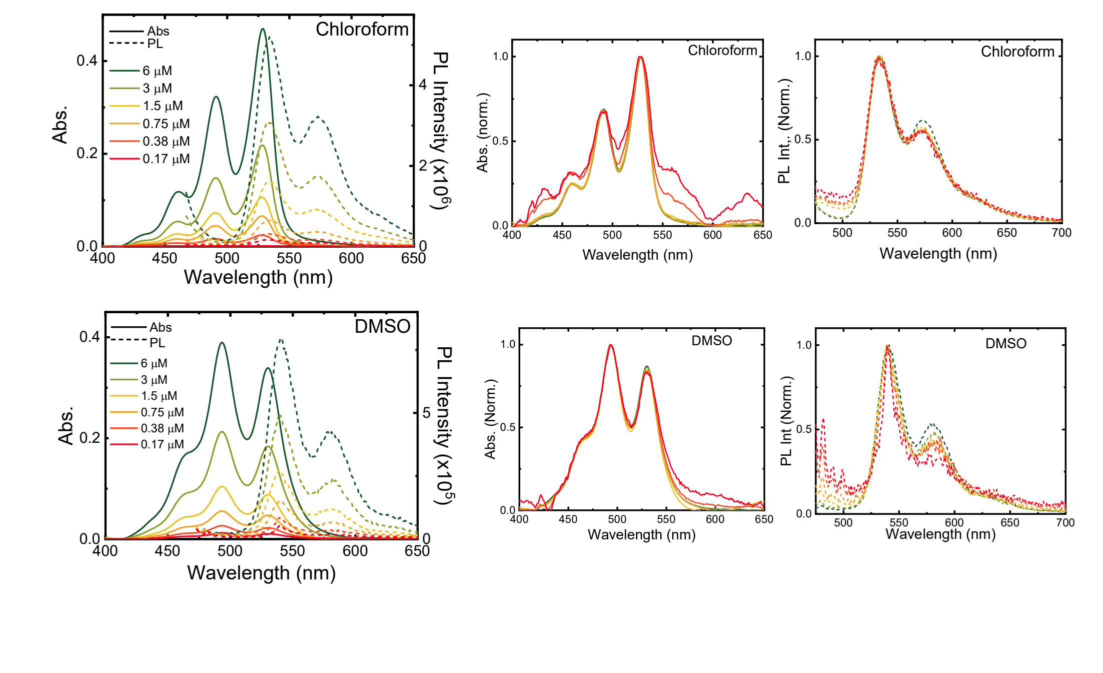

Figure S10. Concentration dependent steady-state UV-vis and PL measurements in both chloroform (open) and DMSO (folded) demonstrate minimal changes upon dilution. This evidences that aggregation effects, both static and dynamic, are due to intramolecular interactions. Note that the increased apparent structure in the spectra at very low (> 0.38 μM) concenrations are due to low signal:noise in these conditions. These measurements taken in a 1 cm pathlength cuvette.

Calculating Free Energies for Charge Separation

Redox Potentials from Cyclic Voltammetry

CV measurements (Figure S11) were carried out in dry dioxane at room temperature using an electrochemical analyser (model CHI620E, CH Instruments). The platinum-inlay working electrode (Beckman M-39273) was polished using a 0.05 μm Alumina water slurry on a felt surface immediately before use. A Pt wire served as an auxiliary electrode, and a saturated calomel electrode (SCE) was used as the reference electrode. Sample concentrations were kept at 10 μM, with 1 mM $TBAPF_6$ as the supporting electrolyte. The solution was purged with dry Ar gas just before each measurement, and the scan rate was 50 mV/s. The oxidation potential of the $AnEt_2$ donor, while a very low signal, is consistent with literature reports on analogous molecules.[3] Similarly, the shift in PDI reduction potential from the open to folded conformation is consistent with earlier work on a PDI folda-dimer.[4]

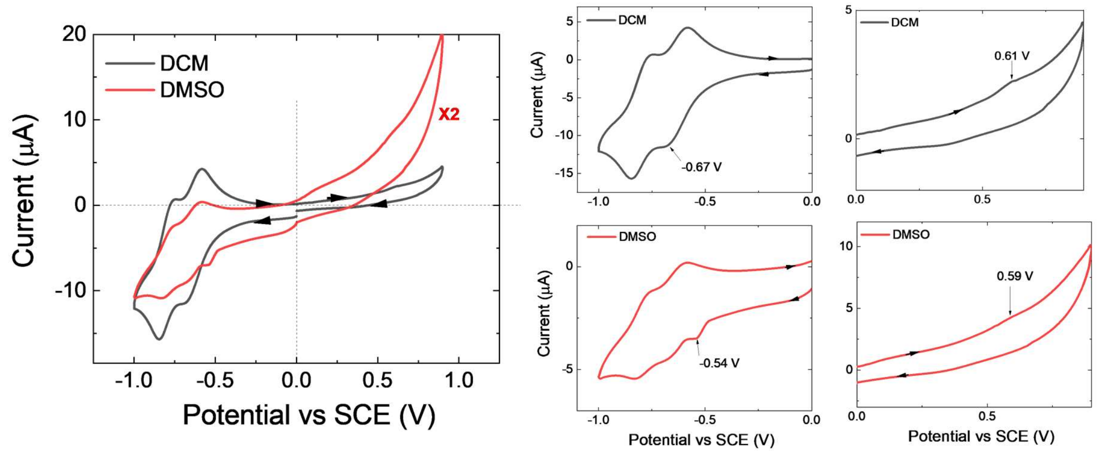

Figure S11. Measured CV of PDI-AnEt$_2$-PDI in open (DCM) and folded (DMSO) conformations. Graphs on the right are zoomed in to the relevant regions where the redox potentials are identified.

Table S2. Redox Values used in Calculations

	Potential (V vs. SCE)	Reference
$E_{ox}(AnEt_2)$	0.61	Measured in this work, Figure S11
E_{red}(PDI) open	-0.65	Measured in this work, Figure S11
E_{red}(PDI) folded	-0.54	Measured in this work, Figure S11
E_{ox}(PDI)	1.61	*J. Am. Chem. Soc.* 2004, 126, 49, 16126–16133

(3) Masui, M.; Sayo, H.; Tsuda, Y. Anodic Oxidation of Amines. Part I. Cyclic Voltammetry of Aliphatic Amines at a Stationary Glassy-Carbon Electrode. *J. Chem. Soc. B* **1968**, No. 0, 973–976. https://doi.org/10.1039/J29680000973.

(4) Samanta, S.; Chaudhuri, D. Suppressing Excimers in H-Aggregates of Perylene Bisimide Folda-Dimer: Role of Dimer Conformation and Competing Assembly Pathways. *Journal of Physical Chemistry Letters* **2017**, *8* (14), 3427–3432. https://doi.org/10.1021/acs.jpclett.7b01338.

Molecular Geometries and Energies

The ground state geometries of the open and folded conformers of PDI-AnEt2-PDI were optimised based on density function theory (DFT) using the wB97X-D function to account for dispersion, as implemented in Gaussian09. Optimisations were carried out using the 6-311G(d,p) basis set in the respective solvents (IEPCM model). The molecular dynamics-based experiments were done with the same basis and function in the gas phase.

Table S3. Optimised distance between donor and acceptor for open and folded form.

**Solvent**	**Energy (eV)**	**$r_{AnEt2\text{-}PBI}$ (Å)**	**$r_{PBI\text{-}PBI}$ (Å)**
Chloroform(open)	-90893.38	9.43	16.70
1,4-Dioxane(open)	-90893.13	9.43	16.69
Toluene(open)	-90893.16	9.43	16.69
DMSO(closed)	-90894.85	9.43	3.63
Acetone(closed)	-90894.79	9.42	3.63
Cyclohexane(closed)	-90894.21	9.41	3.69

Thermodynamic Driving Force using Rehm-Weller expression

The theoretical driving force for the different charge-separation pathways was calculated using the Rehm-Weller expression based on the Born dielectric continuum model:

$$\Delta G_{IP} = e[E_{ox}(D) - E_{red}(A)] - E_{00} \quad (1)$$

$$\Delta G_{CS} = \Delta G_{IP} + C + S \quad (2)$$

$$\Delta G_{CR} = -\Delta G_{IP} \quad (3)$$

$$C = \frac{-e^2}{4\pi\varepsilon_0 r_{DA}\varepsilon_S} \quad (4)$$

$$S = \frac{e^2}{4\pi\varepsilon_0}\left(\frac{1}{2r_D} + \frac{1}{2r_A}\right)\left(\frac{1}{\varepsilon_S} - \frac{1}{\varepsilon_{SP}}\right) \quad (5)$$

- $E_{ox}(D)$ and $E_{red}(A)$ represent the half-wave oxidation potential of donor (D) and acceptor (A), respectively, and are taken from **Error! Reference source not found.**. Note that while the same value of E_{ox} is used for $AnEt_2$ for open and folded form, the value of E_{red} for PDI was experimentally shown to differ in the two geometries.

- E_{00} is the energy of the lowest excited state of the acceptor chromophore and is estimated by the interaction of the normalised absorption and fluorescence spectra. The values used are summarised in the results tables in the main text.
- C and S represent the coulombic interaction and solvation energy of the radical cation and anion molecules.
- r_{DA} is the center-to-center distance between donor and acceptor and is taken from Table S3.
- r_A is the PDI anionic radius, which we took as 7.4 Å from literature. (Guo, et al., J. Am. Chem. Soc, 141, 12789 (2019))
- r_D is the cationic radius. For calculations with $AnEt_2$ as the donor, we used 3.05 Å based on the literature reported value for the structurally similar dimethylaniline (Tavernier, et al., J. Phys. Chem. B 102, 6078 (1998)). For calculations with PDI as donor, we assumed a similar anionic and cationic radius.
- ε_{sp} is the dielectric constant of the solvent used for determining the redox potentials with via electrochemistry.
- ε_s is the dielectric constant of the solvent used in the spectroscopic studies. The values used are summarised in the results tables in the main text.

Table S4. Summary of values used in the calculation for each solvent, along with solvent polarity

	**Solvent**	**Dielectric constant**	**Relative Polarity**	**E_{00}(eV)**
Open	Chloroform	4.81	0.259	2.33
	Dioxane	2.25	0.164	2.35
	Toluene	2.83	0.099	2.33
Folded	DMSO	46.61	0.444	2.31
	Acetone	20.7	0.355	2.37
	Cyclohexane	2.02	0.006	2.39

Figure S12, S13 – Time-Resolved Photoluminsecence (TRPL)

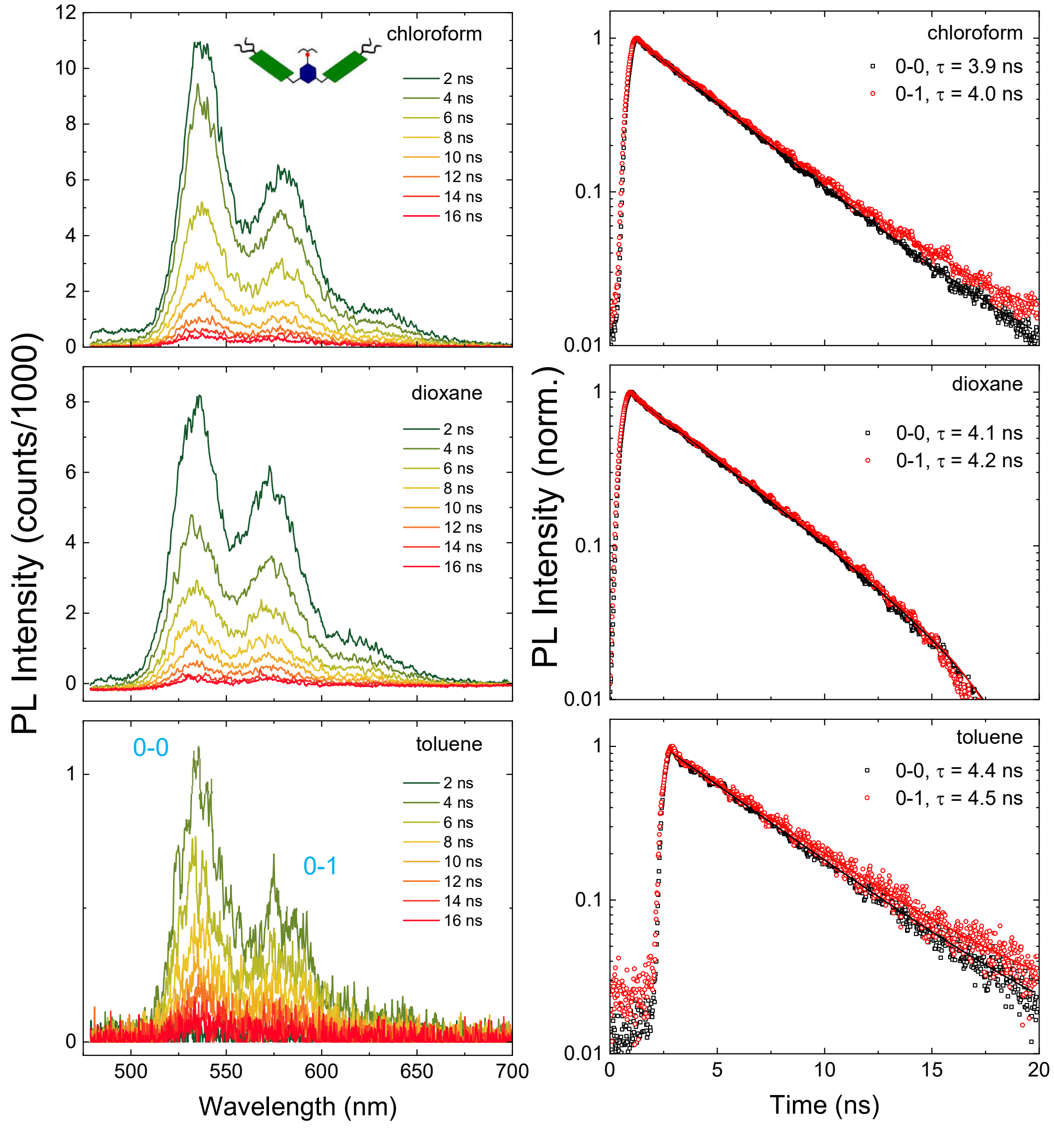

Figure S12. TRPL spectral traces in the open conformation solvents along with transient photoluminescence kinetics monitored at the 0→0 and the 0→1 vibronic bands. Solid lines in kinetics represent monoexponential fits, as summarized in the legend.

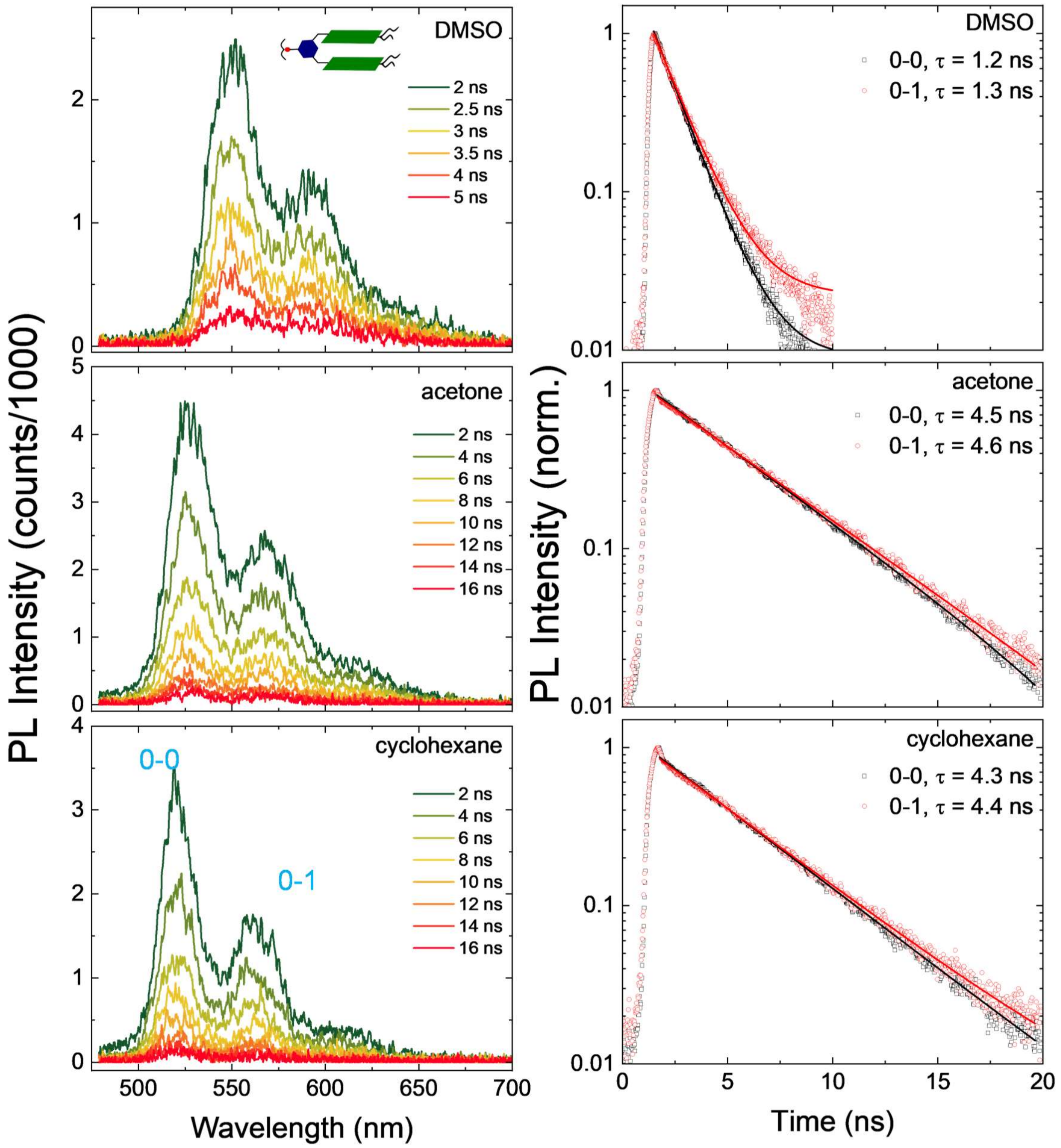

Figure S13. TRPL spectral traces in the folded conformation solvents along with transient photoluminescence kinetics monitored at the 0→0 and the 0→1 vibronic bands. Solid lines in kinetics represent monoexponential fits, as summarized in the legend.

Figure S14 – TA of PDI Ref

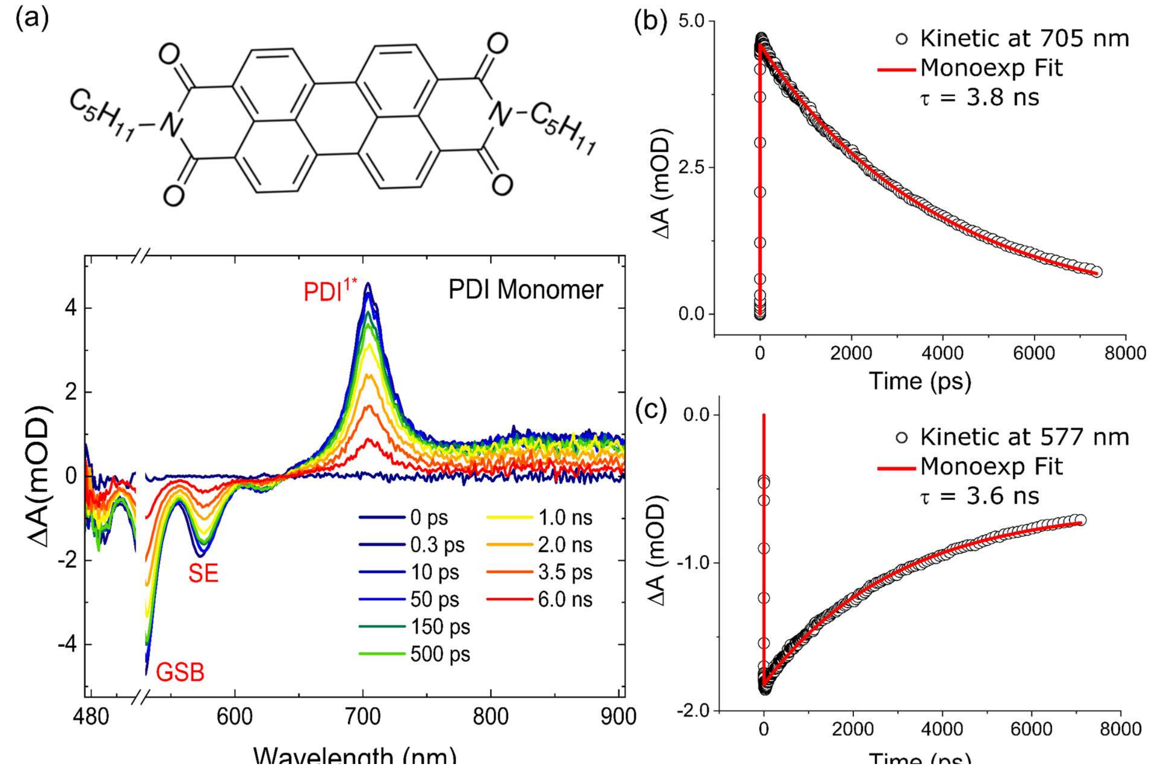

Figure S14. (a) TA measurement of ref PDI monomer in chloroform at 530 nm excitation in a 2mm cuvette. Top right picture shows the chemical structure of PDI monomer used as reference in this study. (b) Transient kinetic of excited state spectra at 705 nm in open black circle with its monoexponential fitting in red. (c) Transient kinetic of stimulated emission at 577 nm in open black circle with its monoexponential decay fit in red.

Figure S15 – Comparing TA lineshapes of PDI-AnEt$_2$-PDI in chloroform and PDI-Ref

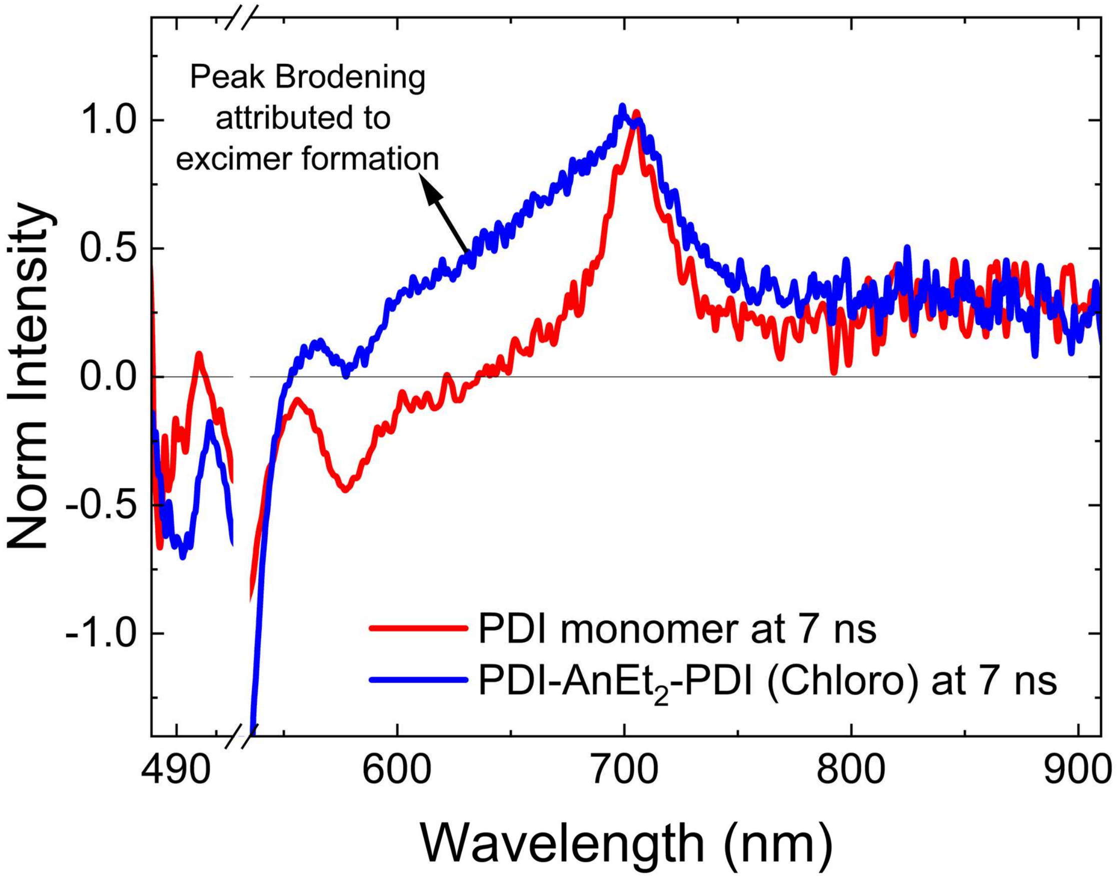

Figure S15: Comparison of TA spectra of PDI-Ref in red and PDI-AnEt$_2$-PDI in chloroform in blue at 7 ns delay time. The spectra is normalized to the difference absorbance value at 705 nm. The transient spectrum of PDI-AnEt2-PDI exhibits a peak broadening in the region between 600-700 nm, consistent with excimer formation.

Figure S16 – TRPL of PDI-AnEt$_2$-PDI in chloroform, 50 ns measurement window

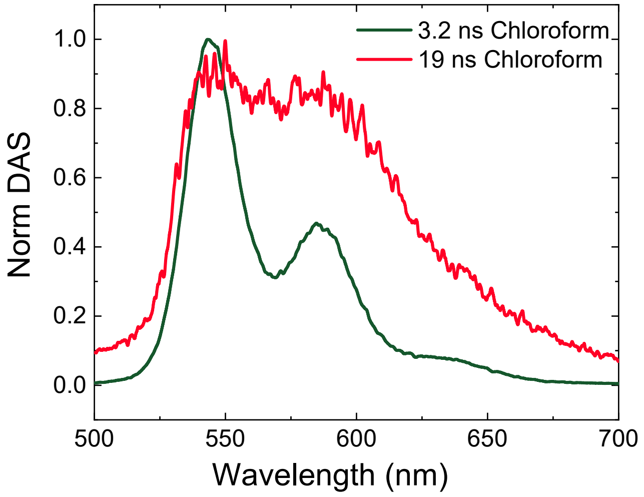

Figure S16. Decay associated spectra from global analysis of PDI-AnEt$_2$-PDI in chloroform, measured with a 50 ns time window. The second component (red) exhibits a 19 ns lifetime and a significant contribution from a red-shifted emission, consistent with an H-type excimer.

Figure S17 – TA of PDI-AnEt$_2$-PDI in protonated dioxane (open form)

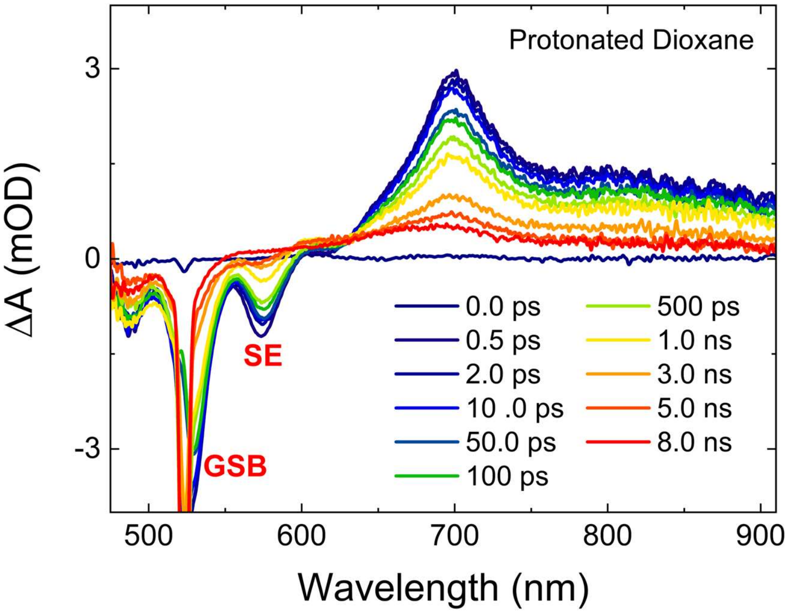

Figure S177. TA measurement of open PDI-AnEt2-PDI in protonated dioxane, λ_{ex} = 530 nm.

Figure S18 – Comparing TA of PDI-AnEt$_2$-PDI in acetone (folded form) at λ_{ex} = 490 and λ_{ex} = 530 nm

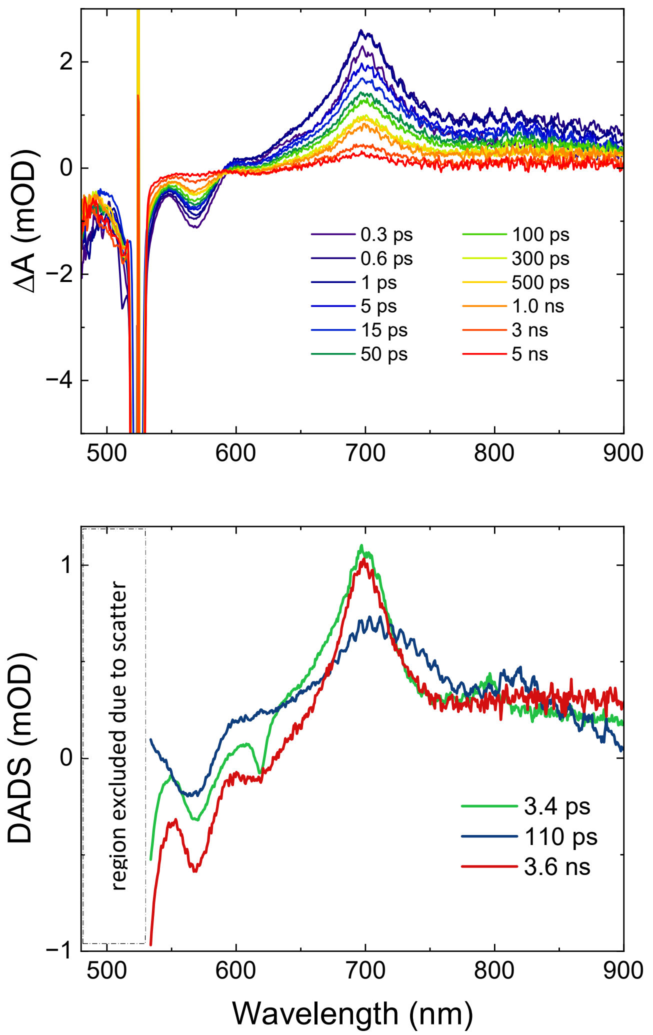

Figure S188. TA spectra of folded PDI-AnEt2-PDI in acetone at (a) λ_{ex} = 530 nm and (b) associated DADS. No clear differences are observed, indicating similar photophysical behavior independent of excitation wavelength. Note that for the DADS the region with the excitation scatter (480-540 nm) was excluded from the fitting.
